# On the problem of confounders in modeling gene expression

**DOI:** 10.1093/bioinformatics/bty674

**Published:** 2018-08-02

**Authors:** Florian Schmidt, Marcel H Schulz

**Affiliations:** 1 High-througput Genomics and Systems Biology, Cluster of Excellence on Multimodal Computing and Interaction, Saarland Informatics Campus, Saarbrücken, Germany; 2 Department of Computational Biology and Applied Algorithmics, Max Planck Institute for Informatics, Saarland Informatics Campus, Saarbrücken, Germany; 3 Graduate School for Computer Science, Saarland Informatics Campus, Saarbrücken, Germany

## Abstract

**Motivation:**

Modeling of Transcription Factor (TF) binding from both ChIP-seq and chromatin accessibility data has become prevalent in computational biology. Several models have been proposed to generate new hypotheses on transcriptional regulation. However, there is no distinct approach to derive TF binding scores from ChIP-seq and open chromatin experiments. Here, we review biases of various scoring approaches and their effects on the interpretation and reliability of predictive gene expression models.

**Results:**

We generated predictive models for gene expression using ChIP-seq and DNase1-seq data from DEEP and ENCODE. Via randomization experiments, we identified confounders in TF gene scores derived from both ChIP-seq and DNase1-seq data. We reviewed correction approaches for both data types, which reduced the influence of identified confounders without harm to model performance. Also, our analyses highlighted further quality control measures, in addition to model performance, that may help to assure model reliability and to avoid misinterpretation in future studies.

**Availability and implementation:**

The software used in this study is available online at https://github.com/SchulzLab/TEPIC.

**Supplementary information:**

Supplementary data are available at *Bioinformatics* online.

## 1 Introduction

Elucidating the mechanism of transcriptional regulation is a major, yet unsolved, task in computational biology. While wet-lab experiments, e.g. knock-outs of Transcription Factors (TFs), can deliver insights into the transcriptional machinery on a causal level (Geier *et al.*, 2007), they are laborious, expensive, and may be inconclusive (Illari and Russo, 2014).

To overcome this issue, several attempts have been made to come up with interpretable models of gene expression using various features as input (Budden *et al.*, 2015; Costa *et al.*, 2011; Li *et al.*, 2015; McLeay *et al.*, 2012; Natarajan *et al.*, 2012; O’Connor and Bailey, 2014; Ouyang *et al.*, 2009; Schmidt *et al.*, 2017; Singh *et al.*, 2016; Wang *et al.*, 2013). These models not only predict gene expression, they also identify a subset of features that can be associated to it. Especially models that are either based on TF ChIP-seq data, or on predicted TF binding events might deliver insights on the overall importance of TFs both within (Ouyang *et al.*, 2009; Schmidt *et al.*, 2017) and between samples (Cheng *et al.*, 2012; Durek *et al.*, 2016; Ouyang *et al.*, 2009). Considering the large amounts of epigenetics data produced in consortia like ENCODE (Dunham *et al.*, 2012), Roadmap (Kundaje *et al.*, 2015), and Blueprint (Adams, 2012), in silico models of transcriptional regulation have become more prevalent.

For example in Ouyang *et al.* (2009), TF ChIP-seq data is used to predict gene expression in mouse embryonic stem cells (mESC) and to assess differential expression between mESCs and embryoid bodies. The authors suggested tissue specific regulators and sketched regulatory roles for several TFs. It was shown that TF-binding sites (TFBS) computed with Position Weight Matrices (PWMs), describing the sequence specificity of TFs, are highly predictive of gene expression and allow close to ChIP-seq quality in terms of model accuracy (Budden *et al.*, 2015; Costa *et al.*, 2011; McLeay *et al.*, 2012; Natarajan *et al.*, 2012; Schmidt *et al.*, 2017). As TF-ChIP-seq data is not available for all TFs in all tissues, these models make use of epigenetics data such as histone marks (HMs) or chromatin accessibility data, e.g. DNase1-seq (Keene *et al.*, 1981) to derive tissue specific TFBS (Cuellar-Partida *et al.*, 2012; Gusmao *et al.*, 2016; Pique-Regi *et al.*, 2011; Schmidt *et al.*, 2017; Sherwood *et al.*, 2014).

A detailed examination of the inferred models revealed that chromatin accessibility data (McLeay *et al.*, 2012), HM abundance, or TF-binding data (Budden *et al.*, 2014) are equally predictive of gene expression, arguing for the presence of shared information. For TF-binding data, it was also shown that there is redundant information between various TFs (Diamanti *et al.*, 2016; Ramachandran *et al.*, 2015; Rye *et al.*, 2011; Yan *et al.*, 2013), which might effect model interpretability and could lead to wrong conclusions about the biological questions at hand (Bessiere *et al.*, 2018). For example, Ramachandran *et al.* (2015) have investigated the redundancy in TF ChIP-seq data and analyzed how it relates to other kinds of epigenetic data such as DNase1-seq and HMs. They argue that only general factors, such as TAF1 or POL2, are universal predictors for gene expression while others do not have more predictive power than chromatin-accessibility. It might be that a part of this redundancy is caused by the plethora of known biases influencing various chromatin profiling experiments, e.g. the so called expression bias of ChIP-seq data (Park *et al.*, 2013) or ChIP-seq antibody quality, PCR amplification biases, sequencing depth, and outlier samples. These biases have been investigated in detail and methods have been suggested to account for them (Diaz *et al.*, 2012; Gusmao *et al.*, 2016; Kuan *et al.*, 2011; Ramachandran *et al.*, 2015; Wang *et al.*, 2017; Yardimci *et al.*, 2014). However, those approaches do not analyze data on a gene-centric level and therefore do not account for biases introduced through data integration, which is the focus of this article. Here, we review confounders in modeling TF-gene scores from both TF ChIP-seq as well as DNase1-seq data and study their effect on gene expression prediction and model interpretation.

## 2 Materials and methods

### 2.1 Data

Here, we use seven paired DNase1-seq and RNA-seq samples obtained from ENCODE and the German epigenomics project (DEEP). Specifically, we use three primary human hepatocyte samples (LiHe1, LiHe2, LiHe3) and one HepG2 sample from DEEP as well as one sample each of K562, GM12878, and H1-hESC cells from ENCODE. From ENCODE, we downloaded quantified gene expression data, DNase1-seq BAM files and narrow peak calls of 33 TF-ChIP-seq experiments for K562, 39 for HepG2, and 50 for both GM12878 and H1-hESCs. We use the hg19 reference genome. A complete list of all ENCODE accession numbers and DEEP sample IDs is provided in Supplementary Table S1. DEEP data can be obtained from EGA (*EGAS00001002073*). Details on DNase1-seq and RNA-seq experimental protocols for DEEP samples are provided in Supplementary Section *Experimental Procedures* of Schmidt *et al.* (2017). Quantification of gene expression from RNA-seq data and peak-calling on DNase1-seq data were performed as described in Supplementary Section S1. For computational annotation of TF binding we use a curated set of 726 position specific energy matrices (PSEMs) obtained from JASPAR (Mathelier *et al.*, 2016), Hocomoco (Kulakovskiy *et al.*, 2016), and the Kellis ENCODE Motif Database (Kheradpour and Kellis, 2014).

### 2.2 Computing TF-gene scores from ChIP-seq data

We compute TF-gene scores $a_{g,t}^{C}$ for gene *g* and TF *t* from TF-ChIP-seq data in three ways:

First, using an exponential decay formulation proposed by Ouyang *et al.* (2009):

(1) $$a_{g,t}^{C}=\sum_{p\in P_{g,50kb}}c_{p,t}e^{-\frac{d_{p,g}}{d_{0}}},$$

where we consider all ChIP-seq peaks *p* in a window of 50 *kb* centered at the most 5′ TSS of genes and sum all ChIP-seq scores $c_{p,t}$ (peak scores computed by the *uniform ENCODE processing pipeline*) weighted by their distance to the TSS $d_{p,g}$. As suggested in Ouyang *et al.* (2009), the parameter *d*_0_ is set to 5000. It controls the intensity of the exponential weight applied to $c_{p,t}$. $P_{g,50kb}$ denotes the set of all peaks around the TSS of gene *g* in the specified window of 50 kb. We refer to these scores as *ChIP-seq TF-features (C)*.

Second, we suggest normalized TF-gene scores ${\bar{a}}_{g,t}^{C}$ (Eq. 2) by dividing $a_{g,t}^{C}$ by the total number of ChIP peaks $c_{g}^{C}$ (Eq. 3) and consider $c_{g}^{C}$ and peak length $l_{g}^{C}$, defined as the total length of all peaks in $P_{g,50kb}$ (Eq. 4), as two additional features:

(2) $${\bar{a}}_{g,t}^{C}=\frac{\sum_{p\in P_{g,50kb}}c_{p,t}e^{-\frac{d_{p,g}}{d_{0}}}}{c_{g}^{C}},$$

(3) $$c_{g}^{C}=\sum_{t\in T}\sum_{p\in P_{g,50kb}}I(c_{p,t})e^{-\frac{d_{p,g}}{d_{0}}},$$

(4) $$l_{g}^{C}=\sum_{t\in T}\sum_{p\in P_{g,50kb}}I(c_{p,t})|p|e^{-\frac{d_{p,g}}{d_{0}}},$$

where T denotes the set of all TFs for which ChIP-seq experiments are available, |p| denotes the length of peak *p*, and *I* is the indicator function. Note that both *c^C^* and *l^C^* are distance weighted too. Normalized scores are denoted by *ChIP-seq TF-features normalized (CN)*. An example is shown in Supplementary Figure S2.

Third, we consider only *c^C^* and *l^C^* as features and refer to those as *ChIP-seq peak-features (CPF)*. By definition (Eq. 3, 4), *c^C^* and *l^C^* capture the regulatory activity in the vicinity of a gene measured with ChIP-seq experiments. Thus, *c^C^* and *l^C^* can be seen as an aggregated view for the activity of transcriptional regulation. An overview on the annotation versions is shown in Table 1.

**Table 1.** Overview on the different score variations of this study

	Abbreviation	Equation	Included features
ChIP-seq TF features	C	(1)	*a^C^*
ChIP-seq TF features normalized	CN	(2)	${\bar{a}}^{C}$
ChIP-seq peak features	CPF	(3, 4)	*c^C^*, *l^C^*
DNase Decay	D	(5)	*a^D^*
DNase Decay-Scaled	DS	(6)	*a^DS^*
DNase Decay normalized	DN	(7, 8, 9)	${\bar{a}}^{D},c^{D},l^{D}$
DNase Decay-Scaled normalized	DSN	(7, 8, 9, 10)	${\bar{a}}^{D},c^{D},l^{D},f^{D}$
DNase peak-features	DPF	(8, 9)	*c^D^*, *l^D^*
DNase peak-features and signal	DPFS	(8, 9, 10)	$c^{D},l^{D},f^{D}$

### 2.3 Computing TF-gene scores from DNase data

The computation of TF-gene scores from DNase1-seq data is conducted with the *TEPIC* approach that also employs the exponential decay formulation by Ouyang *et al.* (2009). Briefly, we compute TF affinities for 726 TFs using TRAP (Roider *et al.*, 2007) in accessible chromatin regions. TF affinities are a quantitative measure of TF binding that can be interpreted as the estimated number of bound molecules to a target site. TRAP computes a score $a_{p,t}$, denoting the TF affinity of TF *t* in DNase1 peak *p* by summing up the contribution of all individual binding sites in *p*. For details see Supplementary Section S3 and Roider *et al.* (2007). In the original TEPIC annotation (termed as *DNase-Decay(D)*), TF-gene scores $a_{g,t}^{D}$ are computed as (Eq. 5)

(5) $$a_{g,t}^{D}=\sum_{p\in P_{g,50kb}}a_{p,t}e^{-\frac{d_{p,g}}{d_{0}}},$$

where P is the set of all considered DNase1 peaks.

The *DNase-Decay-Scaled (DS)* annotation directly integrates the DNase1 signal *s_p_* of peak *p* into the TF-gene score $a_{g,t}^{DS}$ (Eq. 6)

(6) $$a_{g,t}^{DS}=\sum_{p\in P_{g,50kb}}a_{p,t}s_{p}e^{-\frac{d_{p,g}}{d_{0}}}.$$

Here, we propose an extension of the original formulation that (a) accounts for a bias introduced by the length of the open-chromatin peak |p|, which is linked to the definition of TRAP affinities, and (b) provides information on chromatin accessibility in three separate features. The affinities per peak are normalized by the number of possible binding sites |p|−|m|+1 within a peak, where |m| is the length of TF motif *m*, leading to normalized TF-gene scores ${\bar{a}}_{g,t}$ (Eq. 7). Also, we compute three peak-based features per gene: the number of peaks $c_{g}^{D}$ (Eq. 8), the length of peaks $l_{g}^{D}$ (Eq. 9), as well as the aggregated DNase1 signal across all peaks $f_{g}^{D}$ (Eq. 10):

(7) $${\bar{a}}_{g,t}^{D}=\sum_{p\in P_{g,50kb}}\frac{a_{p,t}}{|p|-|m|+1}e^{-\frac{d_{p,g}}{d_{0}}}\;$$

(8) $$c_{g}^{D}=\sum_{p\in P_{g,50kb}}e^{-\frac{d_{p,g}}{d_{0}}},\;$$

(9) $$l_{g}^{D}=\sum_{p\in P_{g,50kb}}|p|e^{-\frac{d_{p,g}}{d_{0}}},$$

(10) $$f_{g}^{D}=\sum_{p\in P_{g,50kb}}s_{p}e^{-\frac{d_{p,g}}{d_{0}}}\;.$$

We refer to the feature set $DN=\{{\bar{a}}^{D},c^{D},l^{D}\}$ as *DNase-Decay-Normalized*, to $DSN=\{{\bar{a}}^{D},c^{D},l^{D},f^{D}\}$ as *DNase-Decay-Scaled-Normalized*, to $DPF=\{c^{D},\;l^{D}\}$ as *DNase peak-features*, and to $DPFS=\{c^{D},l^{D},f^{D}\}$ as *DNase peak-features and signal*.

### 2.4 Linear regression to predict gene expression

As in Schmidt *et al.* (2017), we use linear regression with elastic net penalty implemented in the glmnet R-package (Friedman *et al.*, 2010) to predict gene expression from either *TEPICs* TF-gene scores, or ChIP-seq based TFBS predictions. Elastic net leads to sparse interpretable models and, due to the grouping effect, preserves correlated features, which naturally occur in the problem sets at hand due to co-regulation and co-binding events of TFs. The grouping effect is achieved by combining two regularization terms, the Ridge and the Lasso penalty:

(11) $$\hat{\beta }=\underset{\beta }{\arg min}||y-X\beta |{|}^{2}+\lambda [\alpha ||\beta |{|}^{2}+(1-\alpha )||\beta ||].$$

Here, *β* represents the feature coefficient vector, $\hat{\beta }$ the estimated coefficients, *X* the feature matrix, *y* the response vector, and *λ* regulates the total amount of regularization. The entries of *X* are composed of the features described above, e.g. in case of *C-scores* the rows of *X* contain genes and the columns the TF scores based on ChIP-seq data, thus an entry $X_{g,t}$ corresponds to the TF-gene score $a_{g,t}^{C}$ for gene *g* and TF *t*. Supplementary Section S4 describes the schematics of all used feature matrices.timates, are log-transformed, with a pseudo-count of 1, centered and normalized. Using *X*, we learn a model to predict the gene expression hold in *y*. The parameter *α* controls the trade-off between Ridge and Lasso penalty. It is optimized in a grid search from 0.0 to 1.0 with a step-size of 0.01. The coefficients $\hat{\beta }$ computed by the model can be seen as indicators for the explanatory power of TFs for gene expression. The number of non-zero regression coefficients is denoted with $||\beta |{|}_{0}^{model}$. All results presented in the main figures of this article are based on elastic net regression.

Model performance is assessed on a hold-out test dataset in a ten-fold outer Monte Carlo cross-validation procedure where 80% of the data are randomly selected as training data and 20% as test data. The *λ* parameter regulating the total amount of regularization is fitted in a six-fold inner cross-validation using the *cv.glmnet* procedure. We choose the *λ* achieving the minimum cross validated error, computed as the average mean squared error (MSE) on the inner folds *(lambda.min)*. Final model coefficients are determined according to the selected *λ* and the entire training dataset. The learning procedure is visualized in Supplementary Figure S14.

### 2.5 Row-wise permutation of the feature matrix

To evaluate whether the data contains a systematic bias, we shuffled the original data matrix *X_o_* per gene, i.e. per row, as suggested in (Bessiere *et al.*, 2018), and obtained a randomized matrix *X_r_*. Shuffling the data per gene preserves any confounders affecting all TF scores computed for one gene but eliminates TF specific information. *X_r_* is used as input for the regression throughout this work whenever we refer to permuted data. See Supplementary Section S5 for an example.

### 2.6 Model evaluation using a gold-standard set of gene regulation in primary human hepatocytes

To judge the correctness of TFs that are predicted as tissue-specific regulators, we conduct a comparison against a gold-standard (*GS*) set on primary human hepatocytes. To avoid any biases by a literature defined *GS*, we considered all TFs that are expressed by at least five transcripts per million (TPM) in liver RNA-seq expression data according to the Human Protein Atlas (Uhlen *et al.*, 2015) (www.proteinatlas.org) and are included in our PSEM collection, resulting in a gold-standard set of 200 TFs (c.f. Supplementary Table S2). We compute Precision (Pr) and Recall (Rec) (Supplementary Section S6), where a *True Positive (TP)* is a TF retrieved by the model that is contained in the *GS*, a *False Positive (FP)* is a TF that is inferred by the model but not included in the *GS*, and a *False Negative (FN)* is a TF that is listed in the *GS* but not retrieved by the model. Area under the Precision-Recall(AUPR) curve and PR curves are computed using the *PRROC* package (Grau *et al.*, 2015). In *PRROC*, TFs are sorted according to their regression coefficients.

## 3 Results

**Fig. 1.**
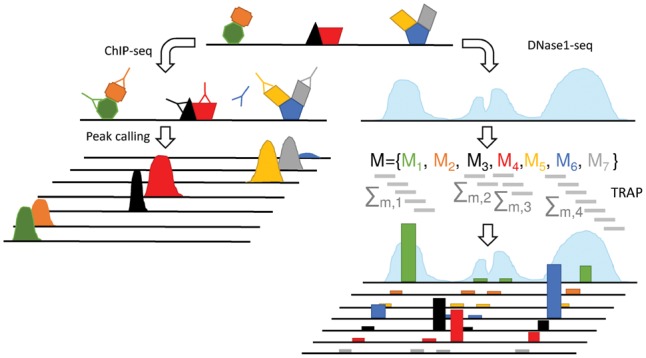
Illustration of the mechanistic differences of TF scores derived from either ChIP-seq or DNase1-seq data. Colored bars indicate TF affinities in DHSs computed with *TRAP* using the PSEMs *M_i_*. While ChIP-seq data can identify TFs acting in complexes, motif based prediction methods screening DHSs are not able to pinpoint these binding events only from sequence data. Identifying TFs that are part of complexes is especially hard in case of indirect binding, i.e. a TF does not bind to the DNA but to another TF. For instance, while the TF colored in orange can be located with ChIP experiments, it remains hidden using predictions based on DNase1 data. Further, the length of a DHS influences motif based scores as the random chance to find a motif hit increases with rising peak length, e.g. although there is no ChIP-seq hit for *M*_1_ in the fourth DHS, we do see a non-zero affinity

To investigate potential biases in TF-gene scores, we analyzed predictive models of gene expression based on either DNase1 or TF-ChIP-seq data. Both models are commonly applied and therefore it is of high relevance to understand potential confounders. As illustrated in Figure 1, the nature of TF binding information retrieved from ChIP-seq experiments is distinct from that of DNase1-seq derived scores in several ways. Firstly, ChIP-seq experiments can be used to identify TFs forming a complex even in case of indirect-binding events, i.e. a TF does not bind the DNA itself but binds to another TF via protein-protein interaction. Such TFs could not be trivially found using DNase1 based prediction methods (Nagy *et al.*, 2016; Wierer and Mann, 2016) that solely rely on motif information, as there might be no binding motif present in the considered genomic loci. Secondly, only the presence of a peak and possibly its intensity are important to compute a TF-gene score from ChIP-seq data. In contrast to that, usually all possible TFBS within a DHS are considered, e.g. in *TRAP* (Manke *et al.*, 2008), therefore the length of a DHS influences the TF scores as longer peaks can obtain a higher score by chance. Due to these differences we deal with both approaches separately.

### 3.1 Aggregated TF-ChIP-seq signal is predictive for gene expression

**Fig. 2.**
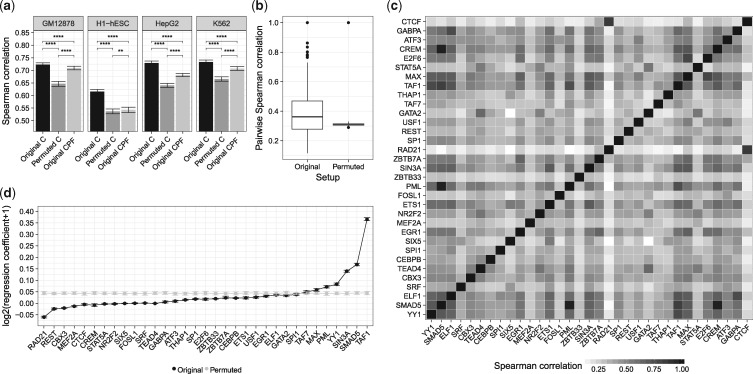
(**a**) The performance of linear regression models predicting gene expression from TF-ChIP-seq data is shown for four different cell lines using the *C* setup with original and per-gene permuted data as well as using the *CPF* scores, which consider only the number and the length of ChIP-peaks aggregated over all TFs. (**b**) Pairwise Spearman correlation of TF-ChIP-seq gene scores computed for 33 TF-ChIP-seq assays in K562. A heatmap of pairwise correlation values for *C* scores is depicted in (**c**). Regression coefficients for the original, not permuted data, and for repeated randomizations are shown in (**d**). Statistical significance in (a) is computed with a Wilcoxon test, where **** refers to a significance level of $10^{-4}$

TF-ChIP-seq data has been shown to be predictive for gene expression (Ouyang *et al.*, 2009; Ramachandran *et al.*, 2015). However, it was observed by Bessiere *et al.* (2018) that per-gene permuted TF-ChIP-seq data has nearly the same predictive power as the original data. We repeated their experiment in a similar fashion and learned linear models with elastic net regularization to predict gene expression in K562, HepG2, GM12878, and H1-hESC cells using ENCODE TF-ChIP-seq data. Although we find that models based on randomized data perform significantly worse compared to the original models (Fig. 2a and Supplementary Fig. S3), their absolute performance is not indicating that the model is based on an erroneous dataset. This suggests that the presence of any TF-ChIP-seq peak in the vicinity of a gene is predictive for gene expression and is supported by the work of Yan *et al.* (2013), who showed that a majority of TF binding in the genome occurs in dense clusters. Thus it is likely that a TF-gene score vector for an expressed gene is not sparse, but holds mostly non-zero values, which might render the scores to be exchangeable without a loss in model performance. We tested this hypothesis, using the *CPF* scoring approach that considers only peak count and peak length per gene. *CPF* models perform worse than the original *C* models, but also better than the permuted *C* models, supporting our hypothesis (Fig. 2a and Supplementary Fig. S3).

To further understand this observation, we computed the pairwise Spearman correlation between all TF-gene scores derived from TF-ChIP-seq data for K562. As shown in Figure 2b, the median correlation between original scores (0.362) is only marginally higher than the correlation on randomized data (0.311). This explains both the good performance of the permuted data and that of the *CPF* scores: the high correlation makes a large portion of the data exchangeable. Nevertheless, the shuffling leads to an obvious loss of several highly correlated TF pairs. To learn about whether these are biologically meaningful correlations, we considered all pairwise correlations as shown in Figure 2c, and indeed found some highly correlated factors to be known interaction partners. For example, CTCF is known to interact with RAD21 (Gosalia *et al.*, 2014) (Spearman correlation: 0.862), or GABPA and ELF1 both belonging to the ETS TF-family (Sharrocks, 2001) (Spearman correlation: 0.776), suggesting that the correlation is at least partially due to cooperativity between TFs.

Bessiere *et al.* (2018) raised concerns that models built from ChIP-seq data might lead to misinterpretations of the regression coefficients, because the models are not robust in randomization experiments. Here, we note that the coefficients learned on the original data are spread over a wide range of values (standard deviation (sd): 0.056), with several coefficients being close to zero. In contrast to that, regression coefficients inferred on randomized data have a small value across all factors (sd: 0.0053) (Fig. 2d). As the regression coefficients are selected stably with low standard deviations across a ten-fold outer cross validation, a wrong interpretation of the models is unlikely. Although the original data is highly correlated, only the coefficients deduced from original data can be meaningfully interpreted, e.g. *TAF1* has the highest regression coefficient. This factor is indispensable to initiate transcription (Bhattacharya *et al.*, 2014), hence it is a good predictor for gene expression.

### 3.2 Accounting for the number of ChIP-seq peaks reduces correlation between TFs

To improve the robustness of ChIP-seq derived TF-gene scores against permutation, we attempted to account for the number of ChIP-seq peaks around a gene’s TSS using the *CN* (Eq. 2) scoring. The new score is motivated by the observation that the feature representing the number of peaks (*c^C^*) has a large, positive regression coefficient in *CPF* models (Supplementary Fig. S1), implying that this quantity itself covers a large portion of the information contained in TF-ChIP-seq data. As shown in Supplementary Figure S2b, the value of *c^C^* is high if there are (i) many TF-ChIP peaks within the considered window and (ii) these peaks are close to the 5′-TSS of the considered gene. Thus, normalizing by *c^C^* leads to a general depletion of TF-gene scores if there are many ChIP peaks present around a gene and simultaneously increases TF-gene scores if there are only a few peaks located in the gene window (Supplementary Fig. S2a and c). Intuitively, this normalization renders individual peaks stronger and weakens peaks within dense clusters.

**Fig. 3.**
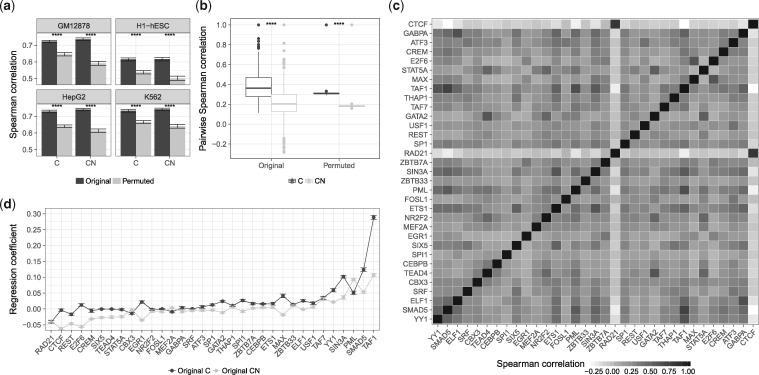
(**a**) Performance of a linear regression model predicting gene expression exploiting the original (*C*) scores is contrasted with the normalized (*CN*) ChIP-seq scores. (**b**) Pairwise Spearman correlation of TF-ChIP-seq gene scores computed for 33 TF-ChIP-seq assays in K562 for *C* and *CN*. (**c**) A heatmap of pairwise correlation values for *CN* scores. Regression coefficients learned for *C*, and *CN* scores are depicted in (**d**). Statistical significance in (a) and (b) is computed with a Wilcoxon test, where **** refers to a significance level of $10^{-4}$

While the permuted data always leads to significantly worse model performance than the original data (Fig. 3a), we find that *CN* scores lead to a significant loss in model performance on permuted data compared to the permuted *C* scores (Supplementary Fig. S3). This indicates that *CN* scores are more robust against permutations than *C* scores, as essential information is lost through the permutation. Simultaneously, model performance on original data is increasing significantly for three out of four samples (Supplementary Fig. S3). The normalization reduces the pairwise correlation between TF-gene scores significantly for original and permuted data, according to a Wilcoxon test (Fig. 3b). Practically, it implies that model performance and pairwise correlation among TF-gene scores could be used to spot errors occurring during data handling or processing rather with *CN* scores than with *C* scores, due to the more pronounced reduction of these measures. Interestingly, the normalization introduced a negative correlation between several TFs (Fig. 3c), for instance between TAF1 and CTCF (–0.282) (Supplementary Fig. S4), which has been reported previously (Kim *et al.*, 2007). Using *C* scores, this pair had a correlation of (0.181), illustrating that the normalization seems to improve modeling the interaction of TFs. Due to the changed correlation between TF features, the regression coefficients for some TFs are altered as well (Fig. 3d). Several TFs that are known to act as a repressor, e.g. E2F6 (Giangrande *et al.*, 2004), REST (Bruce *et al.*, 2006), and EGR1(Arora *et al.*, 2008) obtained a negative regression coefficient using *CN* scores.

### 3.3 Aggregated ChIP-seq peaks indicate regulatory activity

As stated in Section 3.2, the *CPF* scoring (Eq. 3, 4) shows reasonable performance (Fig. 2a). This observation suggests that aggregating ChIP-seq data across several TFs resembles a measure of regulatory activity, which is itself highly predictive for gene expression. This hypothesis can be related to findings by Ramachandran *et al.* (2015). They learned predictive models of gene expression considering only single TF-ChIP seq experiments as input. Additionally, they trained models on DNase1-seq data. They proposed that only a few general TFs are highly predictive for gene expression, while chromatin-accessibility data can replace binding information for most other TFs. We tested this by computing the fraction of ChIP-seq peaks that overlap DNase1-seq peaks in HepG2, K562, GM12878, and in H1-hESCs considering either all, i.e. genome-wide, ChIP-seq peaks or only ChIP-seq peaks in a 50 kb window around the 5′ TSS of all protein coding genes. As shown in Supplementary Figure S7,  71% of all genome-wide ChIP-peaks are covered by a DNase1 peak and even  81% of all ChIP peaks around the TSS of protein coding genes overlap with a DNase1 peak. This indicates that the pure presence of a peak can be seen as an equivalent to the presence of a DHS site, arguing for the exchangeability of TF ChIP-seq data as well as its usage in an aggregated fashion.

### 3.4 Open chromatin characteristics are confounders in predicting TF binding

Although ChIP-seq experiments deliver genome-wide insights into in-vivo TF-binding, it is infeasible to obtain ChIP-seq data for all TFs in all tissues. Therefore, predicting TFBS in open chromatin became a common way to analyze transcriptional regulation through TFs. Next, we examine confounders that affect TF-gene scores calculated from predicted TFBS.

**Fig. 4.**
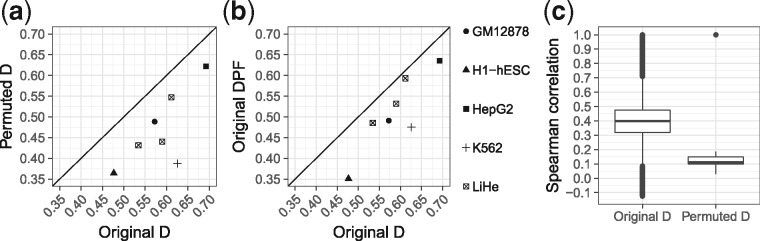
(**a**) Spearman correlation values of linear regression models based on TEPIC scores (*D*) are compared against permuted scores. (**b**) *D* scores are compared against a model using only peak length and peak counts as features (*DPF*). (**c**) Boxplots showing the pairwise Spearman correlation between TF-gene scores, for both original and permuted data across all DNase1 samples using the *D* setup

Using TEPIC TF-gene scores computed according to the *D* setup (Eq. 5), we learned regression models with elastic net regularization to predict gene expression for seven different samples. As reported before (Bessiere *et al.*, 2018), we also find that model performance drops marginally on randomized input (Fig. 4a and Supplementary Fig. S6a–c) and thus renders performance to be inadequate to judge model reliability. To elucidate whether chromatin-accessibility data itself might be a confounder that is inherently contained in TF-gene scores, we compared the performance of a model considering only peak count and peak length per gene as input (*PF*) (Eq. 8, 9) against a model using the full feature matrix (*D*). As shown in Figure 4b (Supplementary Fig. S6d–f), *DPF* models show good performance. Similar observations were made for the *DS* setup (Eq. 6) (Supplementary Fig. S5a and b). As noted by others (McLeay *et al.*, 2012; Ramachandran *et al.*, 2015), this shows that chromatin-accessibility itself is predictive for gene expression. It also supports the idea that TF-gene scores might be linked to chromatin specific features.

In order to follow up on that hypothesis, we computed the pairwise correlation between all TF-gene scores across all genes within each sample. As shown in Figure 4c (Supplementary Fig. S5c for the *DS* setup), some TFs are highly correlated, e.g. TFs with a similar binding motif such as HEY1 and CLOCK, or TEAD1, TEAD3 and TEAD4. Correlation that is due to similar sequence preferences between TFs would be lost in a per-gene randomization. However, correlation that is caused by confounders affecting each gene should not be removed by a per-gene randomization. Therefore, the remaining correlation on permuted data, which is shown in Figure 4c, is likely to be due to confounding variables representing chromatin context introduced while computing the TF-gene scores. Indeed, peak length, peak count, and peak signal are highly correlated to TF affinities (Supplementary Fig. S5d). This is exemplified by Supplementary Figure S10b and c illustrating the correlation between TF-gene scores of HOXA3 and peak length (0.9568) and peak count (0.6786), respectively.

### 3.5 Correcting for confounders improves robustness of TF-gene scores

Due to the computational strategy of how TF scores are computed in the *D* and *DS* setups, namely by summing all possible binding sites in a DHS site, peak length is indirectly incorporated in TF-gene scores. We attempt to correct for this by normalizing TF affinities per DHS by accounting for the number of TFBS (*DN*) (Eq. 7). We apply the same normalization to the *DS* setup and additionally consider the DNase1-seq signal as a separate feature (*DSN*), instead of multiplying it by TF affinities (*DS*).

**Fig. 5.**
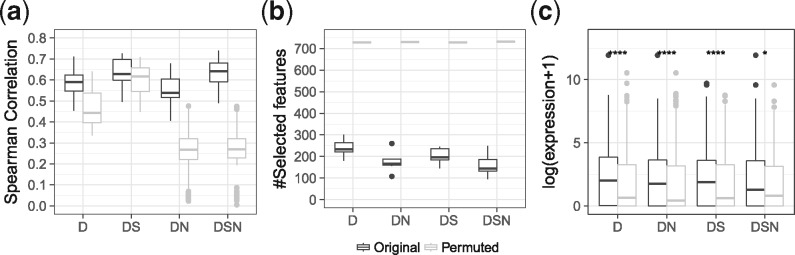
(**a**) This illustrates the performance of gene expression models based on four different annotation setups *(D, DS, DN, DSN)* for original and permuted data. Part (**b**) shows the number of selected features for all annotation variants in original and permuted data using elastic net regularization. Part (**c**) compares the expression of TFs selected by the individual models per setup against the expression of TFs selected on permuted data. According to a Wilcoxon test, the expression differences are significant in all cases with *P *<* *0.05 denoted by *

As shown in Figure 5a, the normalization leads to a significant drop in model performance on permuted data for *DN* and *DSN* (median Spearman correlation 0.268 and 0.269, respectively), while model performance on original data changed only marginally (Supplementary Figs S8a and S9). This holds for elastic net and Lasso regularization (Supplementary Figs S8b and S15). The normalization reduces the correlation between TF-gene scores and chromatin-accessibility features (Supplementary Fig. S10a), e.g. the correlation between TF-gene scores for HOXA3 in LiHe1 and peak length decreased from 0.9568 to 0.5808 (Supplementary Fig. S10b and e). This explains the observed loss in model performance on permuted data. We note that additionally normalizing for peak numbers is not beneficial (Supplementary Fig. S11).

Further, we compared the number of non-zero features derived on permuted and not permuted data observing the general trend $||\beta |{|}_{0}^{D}>||\beta |{|}_{0}^{DS}>||\beta |{|}_{0}^{DN}>||\beta |{|}_{0}^{DSN}$ (Fig. 5b). Strikingly, elastic net constantly selects all features in each annotation setup on permuted data, while Lasso selects only a few representative features (Supplementary Fig. S12). As shown by Zou and Hastie (2005), the grouping effect of the elastic net considers all predictors and assigns them similar regression coefficients if the predictors are part of a group of highly correlated features. Here, all considered features from permuted data form one group of correlated predictors with similar pairwise correlations (Fig. 4c). Therefore, we observe that elastic net selects all features on permuted input and assigns them similar regression coefficients (Supplementary Fig. S13).

### 3.6 Models of gene expression identify expressed TFs as important regulators

Usually, the purpose of gene expression modeling is to infer key regulators by interpreting regression coefficients. Therefore, we checked whether the top 100 TFs selected by the models are expressed, as this could be seen as support for their regulatory role. We considered a TF to be not expressed if it could not be mapped to a gene ID in the expression data. If less than 100 TFs are selected on original data, we choose the same number of TFs from the permuted data. According to a Wilcoxon test, the expression of the top 100 selected TFs derived for each annotation setup on original data is significantly higher than the expression of the top 100 TFs selected on permuted data (Fig. 5c).

### 3.7 Confounder adjustment does not affect model correctness according to AUPR

We performed a detailed evaluation of features for three primary human hepatocyte samples from DEEP using the annotation setups *D, DS, DN*, and *DSN*. The gold standard set contains all TFs that are expressed by at least 5 TPM in liver according to RNA-seq gene expression data obtained from the *Human Protein Atlas*. As outlined in Table 2, the total number of non-zero features varies between annotation versions and samples, while the area under the Precision-Recall curve (AUPR) is similar across annotation setups. In a between sample comparison we do note a drop for LiHe2 with the *DSN* annotation. According to in house quality control, the DNase data for this sample might not be optimal, which could explain the difference to the other hepatocyte replicates. Notably, there is a slight advantage for the unnormalized approaches *D* and *DS* (Supplementary Fig. S16). The differences in the number of selected features could be related to the correlation present in *D* and *DS* scores (Supplementary Fig. S5c and d). As elastic net attempts to find a balance between sparsity and the inclusion of correlated yet predictive features, the number of non-zero features might be higher in *D* and *DS* compared to *DN* and *DSN*. Overall, this analysis does not clearly argue in favor of one of the scoring approaches in terms of biological relevance.

**Table 2.** Number of selected features and AUPRs computed in a gold standard comparison of primary human hepatocytes using elastic net regularization

	#Selected features				AUPR			
	D	DS	DN	DSN	D	DS	DN	DSN
LiHe1	274	210	156	143	0.341	0.360	0.333	0.368
LiHe2	301	145	227	107	0.355	0.346	0.347	0.292
LiHe3	193	297	238	160	0.347	0.333	0.311	0.319

## 4 Discussion and conclusion

Predictive models of gene expression are widely used in computational biology. They allow the integrative analysis of diverse datasets and their interpretation can lead to new hypotheses about molecular processes. In this article, we characterized confounders in TF-gene scores potentially affecting the reliability of such models.

While our analysis showed, similar to the work by Bessiere *et al.* (2018), that row-wise permutation of TF ChIP-seq data does not remove the entire signal, we do find that permuted data leads to models that cannot be interpreted, because the regression coefficients are similar for all TFs. This is due to the ridge penalization incorporated in the elastic net regularization. It distributes the regression weights across correlated features, a behaviour known as the grouping effect (Fig. 5b). The widely used Lasso regularization does not show this useful property on permuted data (Supplementary Fig. S12) and should therefore be used with caution to avoid wrong interpretations. To improve model robustness, we suggest to normalize TF-gene scores by the number of peaks located in the vicinity of a gene’s TSS (*CN*). Thereby, performance of models on permuted data can be lowered, model performance on original data can be increased, and model interpretability is preserved (Fig. 3 and Supplementary Fig. S3). To simplify the detection of cell type specific features further, it would be necessary to include additional cell-type specific enhancer regions, for instance via high-resolution Hi-C data.

Similar to the ChIP-seq data, we find a high pairwise correlation between TF-gene scores derived from chromatin-accessibility data. We identified purely chromatin-accessibility based features, namely peak length, peak count and peak signal as confounding variables (Fig. 4). By accounting for the number of possible TFBS within a DHS, the correlation between the confounders and TF-gene scores could be reduced. Thus, the performance of per-gene randomized input using the normalized data (*DN, DSN*) dropped compared to the original scoring (*D, DS*) (Fig. 5a and Supplementary Fig. S9). Simultaneously, we find only marginal changes in model performance on original input, arguing for the validity of the normalization. Therefore, we suggest to use the normalized scoring, as it helps to pinpoint errors in data handling and eases model interpretation because a smaller number of TFs is selected.

Here, we have used TRAP to compute TF affinities. Instead, any other tool for TFBS prediction could be used as well and would lead to its own distinct biases and corresponding correction approaches, e.g. considering only the most significant motif hit per gene (Wilkins *et al.*, 2016). As explained in Supplementary Section S7, we have also scaled the feature matrices according to the maximum score per gene. Although such a general normalization reduces model performance on permuted data, it also worsens model performance on the actual, not permuted, data (Supplementary Figs S17 and S18), indicating that fine-tuned normalization approaches are required. One obvious question raised by the presented analysis is whether non-linear methods would show a behavior similar to the linear methods. We used Support Vector Regression to answer that question and found that it does not improve prediction accuracy and behaved as the linear methods when applied on permuted input (data not shown).

Notably, no scoring methodology could completely resolve the correlation in TF-gene scores. As illustrated in Figure 1, a complete removal of the correlation should not be expected as the correlation is partially due to biology. For example, ChIP-seq data captures the signal of TFs forming complexes via protein-protein interactions, thereby yielding correlated scores. Also, it is known that TFs tend to bind in clusters (Yan *et al.*, 2013), which is captured by ChIP-seq data and leads to correlated features too. However, the correlation can also be of technical nature, e.g. due to similar binding motifs or open chromatin characteristics. Although we investigated ways how to reduce this correlation, it is inherent, and thus to some extend unavoidable. We like to stress this point and make researchers aware of the potential pitfalls it is causing.

Aside from these analyses, we have illustrated how the number of non-zero features, the magnitude of regression coefficients, and the expression of selected TFs are indicators for model quality and can pinpoint users to potential flaws in feature design or data handling. Importantly, these measures led to the conclusion that results presented in earlier studies using TF ChIP-seq (Ouyang *et al.*, 2009) or predicted TF binding scores (Schmidt *et al.*, 2017) without accounting for confounders are not necessarily incorrect, but highlighted the complexity of prioritizing meaningful TFs due to confounders investigated here. From our perspective the only severe drawback of the earlier scoring methodologies is that potential flaws in modeling cannot be revealed by simply considering model performance in a per-gene randomization. Therefore, researchers should use modeling approaches with caution and be aware of potential confounders.

Common sanity checks as applied here and a sensible choice of the machine learning technique, e.g. elastic net regularization, can help to avoid a wrong interpretation of the models.

## Supplementary Material

Supplementary Material
