# Supplementary material for: On the problem of confounders in modeling gene expression
Source: Bioinformatics. 2018 Aug 2;35(4):711–9. doi: 10.1093/bioinformatics/bty674 (PMC6530814; doi:10.1093/bioinformatics/bty674)
Supplement: Supplementary Material [file bty674_supplementary_material.pdf]

1 Data Processing

Peak calling on DNase1-seq data from DEEP and ENCODE was performed using JAMM (Ibrahim et al., 2015) version 1.0.7.2 using default parameters. All peaks that passed the JAMM filtering step are considered for further usage.

We used bedtools (Quinlan and Hall,2010) version 2.25.0 to generate input bed files for JAMM.

RNA-Seq reads of DEEP data were processed with TopHat 2.0.11 (Trapnell et al., 2009), and aligned with Bowtie 2.2.1 (Langmead and Salzberg, 2012) to NCBI build 37.1 in *--library-type fr-firststrand* and *--b2-very-sensitive* setting.

Gene expression of DEEP data was quantified with Cufflinks version 2.0.2 (Trapnell et al., 2010), and the hg19 reference genome using the options *frag-bias-correct, multi-read-correct*, and *compatible-hits-norm*.

2 Supplementary Tables

**DEEP Sample ID**	**Sample ID used in this study**
01_HepG2_LiHG_Ct1	HepG2
41_Hf01_LiHe_Ct	LiHe1
41_Hf02_LiHe_Ct	LiHe2
41_Hf03_LiHe_Ct	LiHe3
**DEEP File ID**	**Data Type**
01_HepG2_LiHG_Ct1_mRNA_K_1.LXPv1.20150508_genes.fpkm_tracking	Quantified mRNA
01_HepG2_LiHG_Ct1_DNase_S_1.bwa.20140719.bam	Dnase-1 seq
	Quantified mRNA
41_Hf01_LiHe_Ct_DNase_S_1.bwa.20131216.bam	Dnase-1 seq
	Quantified mRNA
41_Hf02_LiHe_Ct_DNase_S_1.bwa.20131216.bam	Dnase-1 seq
	Quantified mRNA
41_Hf03_LiHe_Ct_DNase_S_1.bwa.20150120.bam	Dnase-1 seq
**ENCODE accession number**	**Data Type**
ENCFF000DYC	Quantified mRNA of K562
ENCFF000SVN	DNase -1 seq of K562
ENCFF000CZF	Quantified mRNA of GM12878
ENCFF000SKV	DNase -1 seq of GM12878
ENCFF000SKW	DNase -1 seq of GM12878
ENCFF000SKZ	DNase -1 seq of GM12878
ENCFF000SLB	DNase -1 seq of GM12878
ENCFF000SLD	DNase -1 seq of GM12878
ENCFF000DHQ	Quantified mRNA of H1-hESC
ENCFF000DHS	Quantified mRNA of H1-hESC
ENCFF000DHU	Quantified mRNA of H1-hESC
ENCFF000DHW	Quantified mRNA of H1-hESC
ENCFF000SOA	DNase-1 seq of H1-hESC
ENCFF000SOC	DNase-1 seq of H1-hESC
**ENCODE Accession number**	**TF ChIP-seq in K562**
ENCSR000BNU	ATF3
ENCSR000BRT	CBX3
ENCSR000BRQ	CEBPB
ENCSR077DKV	CREM

ENCSR000DWE	CTCF
ENCSR000BLI	E2F6
ENCSR000BNE	EGR1
ENCSR000BMD	ELF1
ENCSR000BKQ	ETS1
ENCSR000BMV	FOSL1
ENCSR000BLO	GABPA
ENCSR000BKM	GATA2
ENCSR000EFV	MAX
ENCSR000BNV	MEF2A
ENCSR000BRS	NR2F2
ENCSR000BQY	PML
ENCSR000BKV	RAD21
ENCSR000BMW	REST
ENCSR920BLG	SIN3A
ENCSR000BGX	SIX5
ENCSR000FCD	SMAD5
ENCSR000BKO	SP1
ENCSR000BGW	SPI1
ENCSR000BLK	SRF
ENCSR000BRR	STAT5A
ENCSR000BKS	TAF1
ENCSR863KUB	TCF7
ENCSR000BRK	TEAD4
ENCSR000BNN	THAP1
ENCSR000BKT	USF1
ENCSR000BKU	YY1
ENCSR000BKF	ZBTB33
ENCSR000BME	ZBTB7A
	**TF ChIP-seq in HepG2**
ENCFF002CTS	ARID3A
ENCSR000BID	BHLHE40
ENCFF002CTU	BRCA1
ENCFF002CTV	CEBPB
ENCSR000DUG	CTCF
ENCSR000BMZ	ELF1
ENCFF002CUA	ESRRA
ENCSR000ARI	EZH2
ENCSR000BHP	FOSL2
ENCSR000BMO	FOXA1
ENCSR000BNI	FOXA2
ENCSR000BJK	GABPA
ENCSR000BMC	HDAC2
ENCSR000BLF	HNF4A

ENCSR000BNJ	HNF4G
ENCFF002CUD	HSF1
ENCFF002CTY	JUN
ENCSR000BGK	JUND
ENCFF002CUG	MAFF
ENCFF002CUI	MAFK
ENCFF002CUJ	MAX
ENCSR000BQX	NFIC
ENCFF002CUY	NR2C2
ENCFF002CUM	NRF1
ENCSR000BOT	REST
ENCFF002CUT	RFX5
ENCSR00BHU	RXRA
ENCSR000BJX	SP1
ENCSR000BOU	SP2
ENCFF002CUV	SREBF1
ENCFF001VLB	SREBF2
ENCSR000BLV	SRF
ENCSR000BJN	TAF1
ENCFF002CUW	TBP
ENCSR200BJG	TCF12
ENCFF002CUX	TCF7L2
ENCSR000BGM	USF1
ENCFF002CUZ	USF2
ENCSR000BHR	ZBTB33
	**TF ChIP-seq in H1-hESC**
ENCFF002CIR	ATF2
ENCFF002CIS	ATF3
ENCFF002CQP	BACH1
ENCFF002CIT	BCL11A
ENCFF002CQQ	BRCA1
ENCFF002CQR	CEBPB
ENCFF002CQS	CHD1
ENCFF002CQT	CHD2
ENCFF002CQW	CTBP2
ENCFF002CIU	CTCF
ENCFF002CIV	EGR1
ENCFF002CJC	EP300
ENCFF002CDT	EZH2
ENCFF002CIW	FOSL1
ENCFF002CIX	GABPA
ENCFF002CQX	GTF2F1
ENCFF002CIY	HDAC2
ENCFF002CQU	JUN

ENCFF002CQY	JUND
ENCFF002CDU	KDM5A
ENCFF002CQZ	MAFK
ENCFF002CRA	MAX
ENCFF002CRB	MXI1
ENCFF002CQV	MYC
ENCFF002CJA	NANOG
ENCFF002CRC	NRF1
ENCFF002CJE	POLR2A
ENCFF002CJF	POU5F1
ENCFF002CRD	RAD21
ENCFF002CJG	RAD21
ENCFF002CDV	RBBP5
ENCFF002CJB	REST
ENCFF002CRE	RFX5
ENCFF002CJH	RXRA
ENCFF002CRF	SIN3A
ENCFF002CJJ	SIX5
ENCFF002CJK	SP1
ENCFF002CJL	SP2
ENCFF002CJM	SP4
ENCFF002CJN	SRF
ENCFF002CRG	SUZ12
ENCFF002CJO	TAF1
ENCFF002CJP	TAF7
ENCFF002CRH	TBP
ENCFF002CJQ	TCF12
ENCFF002CJR	TEAD4
ENCFF002CJS	USF1
ENCFF002CRI	USF2
ENCFF002CJT	YY1
ENCFF002CRJ	ZNF143
	**TF ChIP-seq in GM12878**
ENCFF002CGO	ATF2
ENCFF002CGP	ATF3
ENCFF002CGQ	BATF
ENCFF002CGR	BCL11A
ENCFF002CGS	BCL3
ENCFF002CGT	BCLAF1
ENCFF809BIO	CBFB
ENCFF002CGU	CEBPB
ENCFF804OVD	CREM
ENCFF002CGV	EBF1
ENCFF515PNJ	EED

ENCFF002CGW	EGR1
ENCFF002CGX	ELF1
ENCFF002CHI	EP300
ENCFF002CGY	ETS1
ENCFF191HSP	ETV6
ENCFF002CGZ	FOXM1
ENCFF002CHA	GABPA
ENCFF002CHB	IRF4
ENCFF939TZS	JUNB
ENCFF002CHC	MEF2A
ENCFF002CHD	MEF2C
ENCFF002CHE	MTA3
ENCFF002CHF	NFATC1
ENCFF002CHG	NFIC
ENCFF002CHJ	PAX5
ENCFF002CHK	PAX5
ENCFF002CHL	PBX3
ENCFF002CHM	PML
ENCFF002CHO	POLR2A
ENCFF002CHP	POU2F2
ENCFF002CHR	RAD21
ENCFF002CHH	REST
ENCFF002CHS	RUNX3
ENCFF002CHT	RXRA
ENCFF002CHU	SIX5
ENCFF374VLY	SMAD5
ENCFF002CHV	SP1
ENCFF002CHQ	SPI1
ENCFF002CHW	SRF
ENCFF002CHX	STAT5A
ENCFF002CHY	TAF1
ENCFF002CHZ	TCF12
ENCFF002CIA	TCF3
ENCFF144PGS	TCF7
ENCFF002CIB	USF1
ENCFF002CIC	YY1
ENCFF694OTE	ZBED1
ENCFF002CID	ZBTB33
ENCFF002CIE	ZEB1

Supplementary Table 1: IDs of ENCODE and DEEP data used in this study.

ID2	E2F4	MAX	CEBPB	SREBF2	NR3C1	CEBPZ	TOPORS
GATA4	ELK1	TBX15	SRF	ETS1	ARNT	MAZ	HERPUD1
HSF1	ZBTB18	CENPB	TGIF1	YY1	NFIX	SMAD2	CDC5L
ESR1	HES1	CEBPD	RFX5	SPI1	ELF2	NR4A1	HMGN3
CTCF	NFATC3	SOX5	SP3	IRF8	FUBP1	NR1D1	CCNT2
RARA	ELK4	NR2F6	USF1	SP1	TFDP1	PBX2	RAD21
IRF1	FOSL2	ZBED1	MEF2A	ESRRA	PBX3	GATA6	SETDB1
STAT6	RXRA	FOXO1	NFE2L2	KLF4	NR4A3	HMGA1	GTF2I
MYC	TCF12	JUNB	ZFX	NFKB2	BACH1	NR1H2	HBP1
CREB1	NR5A2	FOXO3	ZNF410	PPARG	PPARA	FOSB	PTEN
STAT3	BHLHE40	GABPA	HNF4A	ELK3	MBD2	ETS2	THRB
ATF4	JUND	RELA	DBP	FOXJ3	EPAS1	KLF6	CCDC6
ERF	JDP2	NFYA	NFIA	EGR1	NR1H4	SMAD4	HDAC2
TP53	HNF4G	CREB3	MLX	IRF2	NFYC	STAT2	HLX
HNF1A	CUX2	ZNF263	ELF3	SMAD3	HIF1A	TRIM28	NFE2L1
MEF2C	USF2	AR	FOS	SNAI2	DDIT3	NFIB	CHD2
KLF12	SREBF1	HLF	ZEB1	ELF1	AHR	SMC3	ARHGEF12
FOXA1	REST	NFKB1	RORA	TCF4	NR1I2	NR2F2	MXI1
NR2F1	IRF9	NFIC	RREB1	CREM	BPTF	IRF6	SIN3A
CREB3L2	JUN	CEBPA	ZNF143	XBP1	SMAD1	ZNF384	ZBTB16
BCL6	TEAD1	NFIL3	MLXIPL	STAT1	FOXA3	BBX	SP100
ATF1	ATF7	TFCP2	TEF	FOXA2	NR1I3	EP300	PATZ1
CEBPG	HLTF	NR4A2	ATF3	ONECUT1	MAF	ZBTB14	ITGB2
NFYB	ZBTB7B	MAFF	ZBTB33	FOXP1	ATF2	ZNF281	ZNF691
PROX1	CUX1	MAFB	TCF7L2	GRHL1	IRF3	RBPJ	ATF6

Supplementary Table 2: IDs of TFs used in a gold standard comparison for regulation in primary human hepatocytes

3 Details on TRAP

Extensive details on the mathematical background of TRAP can be found in Roider et al. (Bioinformatics, 2007). Here, we only provide a brief summary of Section 2.3 of the aforementioned paper.

In TRAP, one assumes that the fraction of TFs bound to a certain genomic location S is at an equilibrium such that the fraction of bound sites *p(S)* can be denoted as

$$p(S) = \frac{K(S)*[TF]}{1+K(S)*[TF]}.$$

Here *K* denotes a site-specific equilibrium constant, which depends on the site with highest affinity (S_0), a TF specific mismatch energy *E(S)* and the Boltzmann constant k_B:

$$K(S) = K(S_0)e^{-\beta E(S)}$$

Thus, we can denote p(s) as:

$$p(S) = \frac{K(S_0)*[TF]*e^{-\beta E(S)}}{1+K(S_0)*[TF]*e^{-\beta E(S)}} = \frac{R_0*e^{-\beta E(S)}}{1+R_0*e^{-\beta E(S)}}.$$

The mismatch energy *E(S)* is computed using a TF motif matrix according to:

$$\beta E(S) = \frac{1}{\lambda}\sum_{i=1}^{W}\sum_{\alpha=A,C,G,T} S_i^{\alpha} \log(\frac{m_{i,max}}{m_{i,\alpha}} b_{i,\alpha}).$$

Here S_i^{α}, is an indicator function evaluating to 1 if the considered sequence S has letter α at position *i*. The most frequent element in the motif matrix is denoted by $m_{i,max}$. λ is a parameter used to scale the mismatch energy.

Thus, there are only two sequence and TF-independent parameters R_0 and λ. For details on how these parameters are determined, please consult Sections 2.3 and 3.1 of Roider et al. (Bioinformatics, 2007).

Overall, TRAP computes the expected number *N* of TFs bound to sequence *s* with length *l* by summing up the binding score for each individual binding site in *s*:

$$N = p(S) = \sum_{l=1}^{L-W} p_l = \sum_{l=1}^{L-W} \frac{R_0*e^{-\beta E_l(\lambda)}}{1+R_0*e^{-\beta E_l(\lambda)}}.$$

Here, W denotes the length of the motif for the TF of interest.

4 Schematics of feature matrices

In the following, we sketch the content of the feature matrices used for the linear regression setups depending on the used annotation version. Note that the gene expression used as response is not contained in the feature matrix.

4.1 ChIP-seq TF features (C)

	Chipped TF 1	...	Chipped TF n
**Gene 1**	$a_{1,1}^{C}$		$a_{1,n}^{C}$
**...**			
**Gene m**	$a_{m,1}^{C}$		$a_{m,n}^{C}$

4.2 ChIP-seq TF features normalized (CN)

	Chipped TF 1	...	Chipped TF n
**Gene 1**	$\bar{a}_{1,1}^{C}$		$\bar{a}_{1,n}^{C}$
**...**			
**Gene m**	$\bar{a}_{m,1}^{C}$		$\bar{a}_{m,n}^{C}$

4.3 ChIP-seq peak features (CPF)

	ChIP peak count	ChIP peak length
**Gene 1**	c_{1}^{C}	l_{1}^{C}
**...**		
**Gene m**	c_{m}^{C}	l_{m}^{C}

4.4 DNase-Decay (D) and DNase-Decay-Scaled (DS)

	Predicted TF 1	...	Predicted TF n
**Gene 1**	$a_{1,1}^{\mathrm{D(S)}}$		$a_{1,n}^{\mathrm{D(S)}}$
**...**			
**Gene m**	$a_{m,1}^{\mathrm{D(S)}}$		$a_{m,n}^{\mathrm{D(S)}}$

4.5 DNase-Decay normalized (DN)

	Predicted TF 1	...	Predicted TF n	Peak count DNase	Peak length DNase
**Gene 1**	$\bar{a}_{1,1}^{D}$		$\bar{a}_{1,n}^{D}$	c_{1}^{D}	l_{1}^{D}
**...**					
**Gene m**	$\bar{a}_{m,1}^{D}$		$\bar{a}_{m,n}^{D}$	c_{m}^{D}	l_{m}^{D}

4.6 DNase peak-features (DPF)

	Peak count DNase	Peak length DNase
**Gene 1**	c_{1}^{D}	l_{1}^{D}
**...**		
**Gene m**	c_{m}^{D}	l_{m}^{D}

4.7 DNase peak-features and signal (DPFS)

	Peak count DNase	Peak length DNase	Peak signal DNase
**Gene 1**	c_1^D	l_1^D	f_1^D
**...**			
**Gene m**	c_m^D	l_m^D	f_m^D

4.8 DNase-Decay-Scaled normalized (DSN)

	Predicted TF 1	...	Predicted TF n	Peak count DNase	Peak length DNase	Peak signal DNase
**Gene 1**	$\bar{a}_{1,1}^D$		$\bar{a}_{1,n}^D$	c_1^D	l_1^D	f_1^D
**...**						
**Gene m**	$\bar{a}_{m,1}^D$		$\bar{a}_{m,n}^D$	c_m^D	l_m^D	f_m^D

5 Example for the permutation of the feature matrix

In this article we follow the permutation strategy suggested by Bessiere et al. (PLoS Comput. Biol., 2018). They suggested to randomize the feature matrix independently for each row, i.e. per gene. Thereby TF specific signal would be lost, but confounders that affect all scores for one gene would be preserved. In the following example, the color code visualizes the effect of the permutation.

	TF 1	TF 2	TF 3	...
**Gene 1**	$a_{1,1}$	$a_{1,2}$	$a_{1,3}$	
**Gene 2**	$a_{2,1}$	$a_{2,2}$	$a_{2,3}$	
**Gene 3**	$a_{3,1}$	$a_{3,2}$	$a_{3,3}$	
**...**				

Permute →

	TF 1	TF 2	TF 3	...
**Gene 1**	$a_{1,3}$	$a_{1,2}$	$a_{1,1}$	
**Gene 2**	$a_{2,2}$	$a_{2,3}$	$a_{2,1}$	
**Gene 3**	$a_{3,1}$	$a_{3,3}$	$a_{3,2}$	
**...**				

6 Precision (Pr) and Recall (Rec) Computation

Precision (PR) and Recall (Rec) are computed from True Positives (TP), False Positives (FP) and False Negatives (FN) as:

$$\mathrm{Pr} = \frac{TP}{TP + FP}$$

$$Rec = \frac{TP}{TP + FN}$$

7 Scaling feature matrices per row (i.e. per gene)

In addition to the feature matrices discussed in the main paper, which are listed in Sup. Section 4, we tested the performance of feature matrices that are scaled according to the maximum value per row (i.e. per gene). In the DNase1 case the scaled score $\tilde{a}^D_{g,t}$ for gene g and TF t is computed according to

$$\tilde{a}^D_{g,t} = \frac{a^D_{g,t}}{\max_{t \in T}(a^D_{g,t})}.$$

We refer to $\tilde{a}^D_{g,t}$ as *maximized D* scores.

Comparably, scaled DS scores are computed as

$$\tilde{a}^{DS}_{g,t} = \frac{a^{DS}_{g,t}}{\max_{t \in T}(a^{DS}_{g,t})},$$

which we refer to as *maximized DS* scores.

The corresponding feature matrices are:

	**Predicted TF 1**	**...**	**Predicted TF n**
**Gene 1**	$\tilde{a}^{\mathrm{D(S)}}_{1,1}$		$\tilde{a}^{\mathrm{D(S)}}_{1,n}$
**...**			
**Gene m**	$\tilde{a}^{\mathrm{D(S)}}_{m,1}$		$\tilde{a}^{\mathrm{D(S)}}_{m,n}$

In the ChIP-seq case, we compute scaled ChIP-seq scores, abbreviated with *CM*, as

$$\tilde{a}^C_{g,t} = \frac{a^C_{g,t}}{\max_{t \in T}(a^C_{g,t})}.$$

Here, the corresponding feature matrix can be sketched as:

	**Chipped TF 1**	**...**	**Chipped TF n**
**Gene 1**	$\tilde{a}^C_{1,1}$		$\tilde{a}^C_{1,n}$
**...**			
**Gene m**	$\tilde{a}^C_{m,1}$		$\tilde{a}^C_{m,n}$

Note that we do not additionally consider scaling per column (i.e. per TF), because the feature matrices are already scaled per column in our regression setup. Results based on these scores are shown in Sup. Figures. 18 and 19.

8 Supplementary Figures

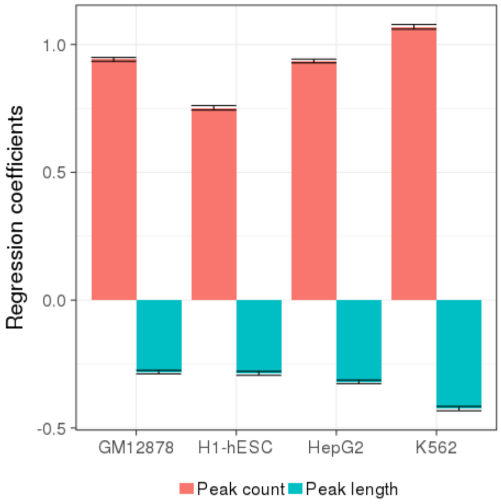

Supplementary Figure 1: This Figure depicts the regression coefficients of *Peak count* and *Peak length* in models using only peak features derived from TF-ChIP-seq data of GM12878, H1-hESC, HepG2, and K562.

(a) $a_{g,t}^{C} = \sum_{p \in P_{g,50kb}} c_{p,t} e^{-\frac{d_{p,g}}{d_0}}$

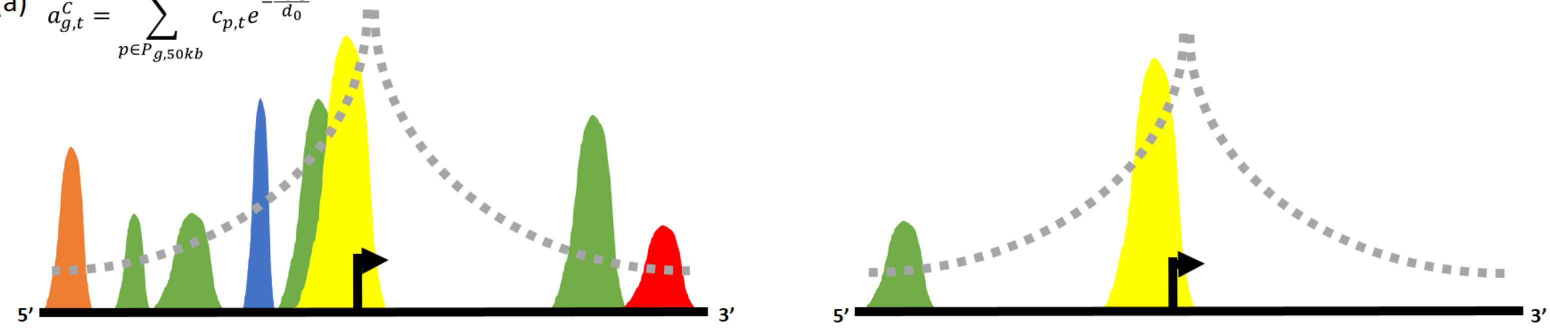

$a_{g1,orange}^{C} = 90 \cdot 0.001 = 0.09$ $\quad a_{g1,blue}^{C} = 100 \cdot 0.5 = 50$ $\quad a_{g1,yellow}^{C} = 120 \cdot 0.9 = 108$ $\quad a_{g1,red}^{C} = 40 \cdot 0.001 = 0.04$

$a_{g2,green}^{C} = 50 \cdot 0.001 = 0.05$ $\quad a_{g2,yellow}^{C} = 120 \cdot 0.85 = 102$

$$a_{g1,green}^{C} = 50 \cdot 0.05 + 50 \cdot 0.1 + 100 \cdot 0.8 + 100 \cdot 0.05 = 2.5 + 5 + 80 + 5 = 92.5$$

(b) $c_{g}^{C} = \sum_{t \in T} \sum_{p \in P_{g,50kb}} I(c_{p,t}) e^{-\frac{d_{p,g}}{d_0}}$

$$c_{g1}^{C} = 0.001 + 0.05 + 0.1 + 0.5 + 0.8 + 0.9 + 0.05 + 0.001 = 2.402$$

$$c_{g2}^{C} = 0.001 + 0.85 = 0.851$$

(c) $\bar{a}_{g,t}^{C} = \frac{a_{g,t}^{C}}{c_{g}^{C}}$

$\bar{a}_{g1,orange}^{C} = \frac{0.09}{2.402} = 0.0375$ $\quad \bar{a}_{g1,blue}^{C} = \frac{50}{2.402} = 20.82$ $\quad \bar{a}_{g1,yellow}^{C} = \frac{108}{2.402} = 44.96$ $\quad \bar{a}_{g2,green}^{C} = \frac{0.05}{0.851} = 0.059$ $\quad \bar{a}_{g2,yellow}^{C} = \frac{102}{0.851} = 119.86$

$\bar{a}_{g1,green}^{C} = \frac{92.5}{2.402} = 38.51$ $\quad \bar{a}_{g1,red}^{C} = \frac{0.04}{2.402} = 0.017$

Supplementary Figure 2: (a), the computation of the TF-gene scores $a_{g,t}^{C}$ from ChIP-seq data is shown for two genes g_1 and g_2 as well as for several TFs. In (b), we show how the normalization factor c_{g}^{C} is computed. Part (c) illustrates how the normalized TF-gene scores are computed. As one can see, the scores for g_2 are increasing, as there are only very few peaks in the vicinity of that gene. Simultaneously all scores of g_1 are shrinked.

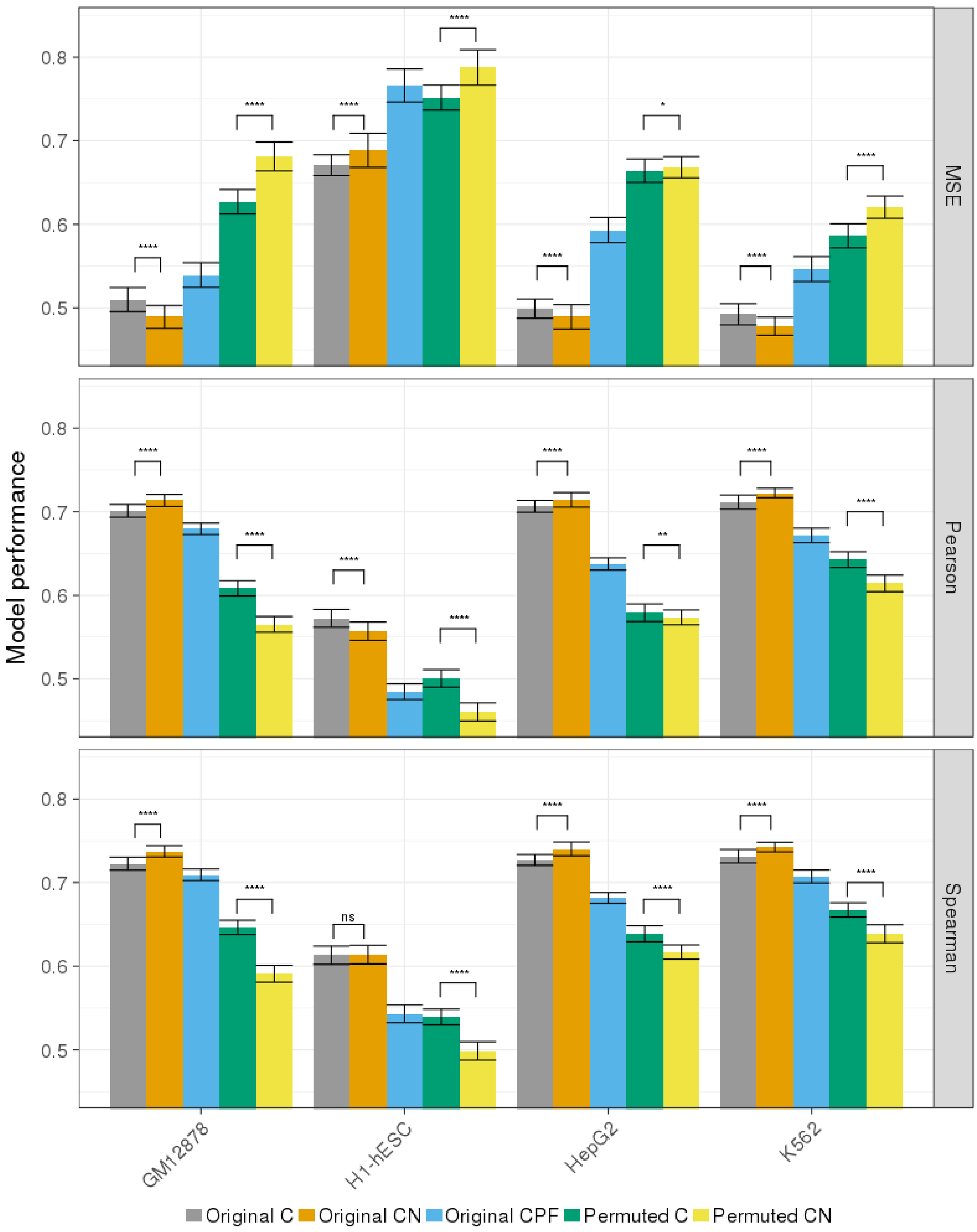

Supplementary Figure 3: Here, we show mean squared error (MSE), Spearman and Pearson correlation for all considered ChIP-seq based models and all available cell-lines.

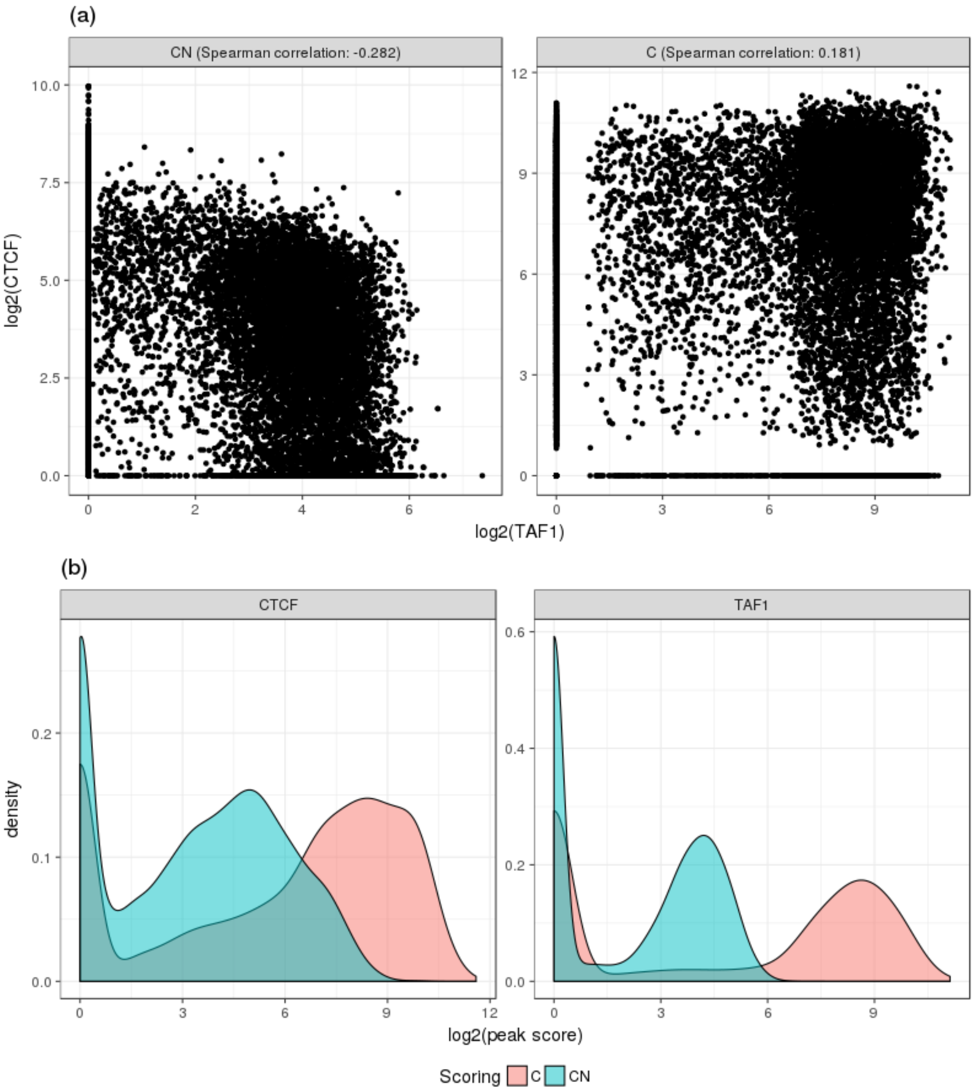

Supplementary Figure 4: (a) shows the changed correlation between TAF1 and CTCF comparing C and CN scores. (b) Indicates the changes in the distribution of peak scores due to the normalization.

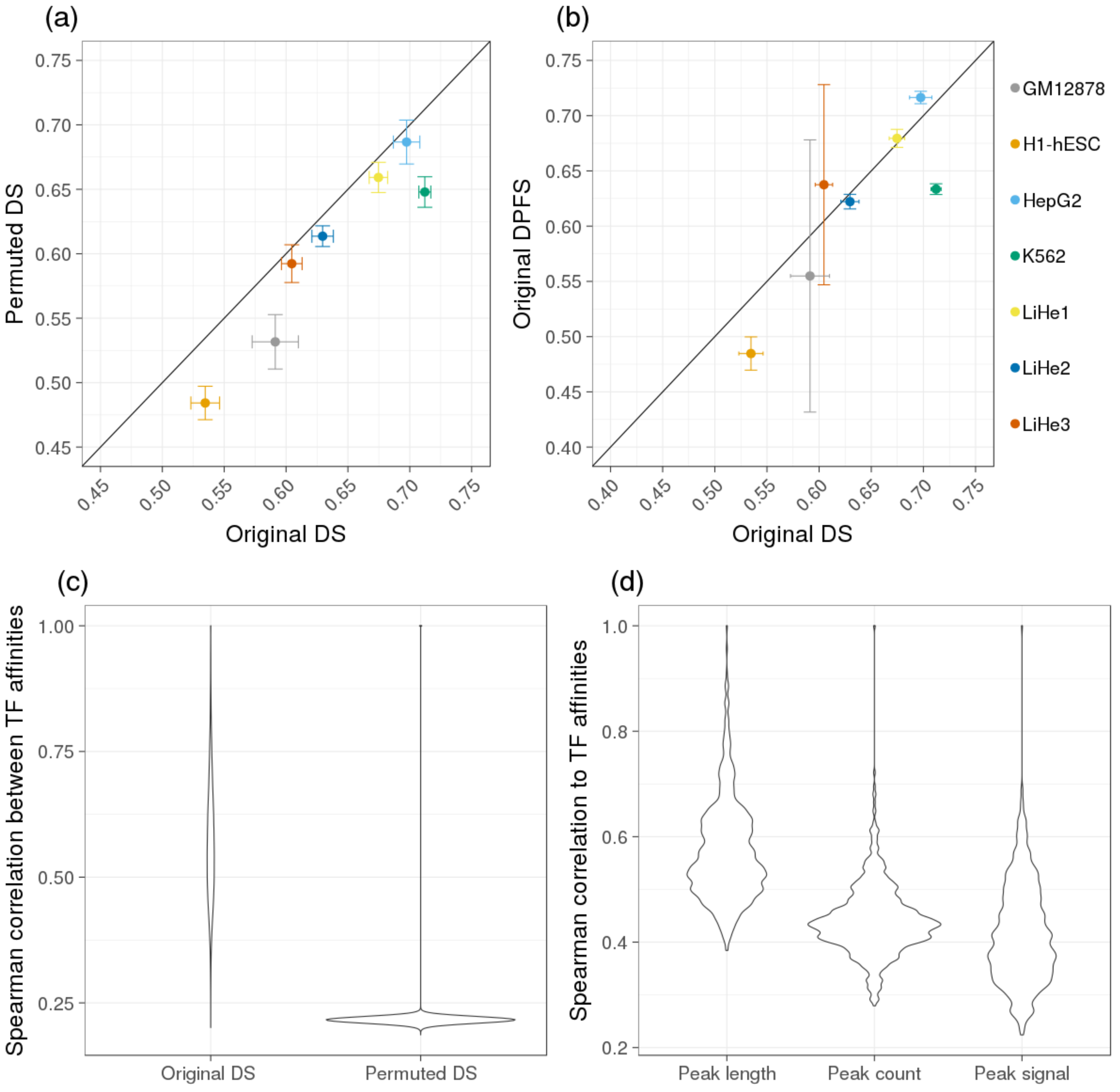

Supplementary Figure 5: Figure 5a contrasts model performance, measured with Spearman correlation, using DS scores on original and permuted data. Figure 5b compares the original DS feature space against the extended feature space with features for *Peak count, Peak length* and *Peak signal,* also in terms of Spearman correlation. Figure 5c shows all pairwise correlations of TF affinities on both original and permuted data. Figure 5d depicts the pairwise correlation of TF affinities against *Peak length, Peak count*, and *Peak signal*. All Figures are based on elastic net regularization.

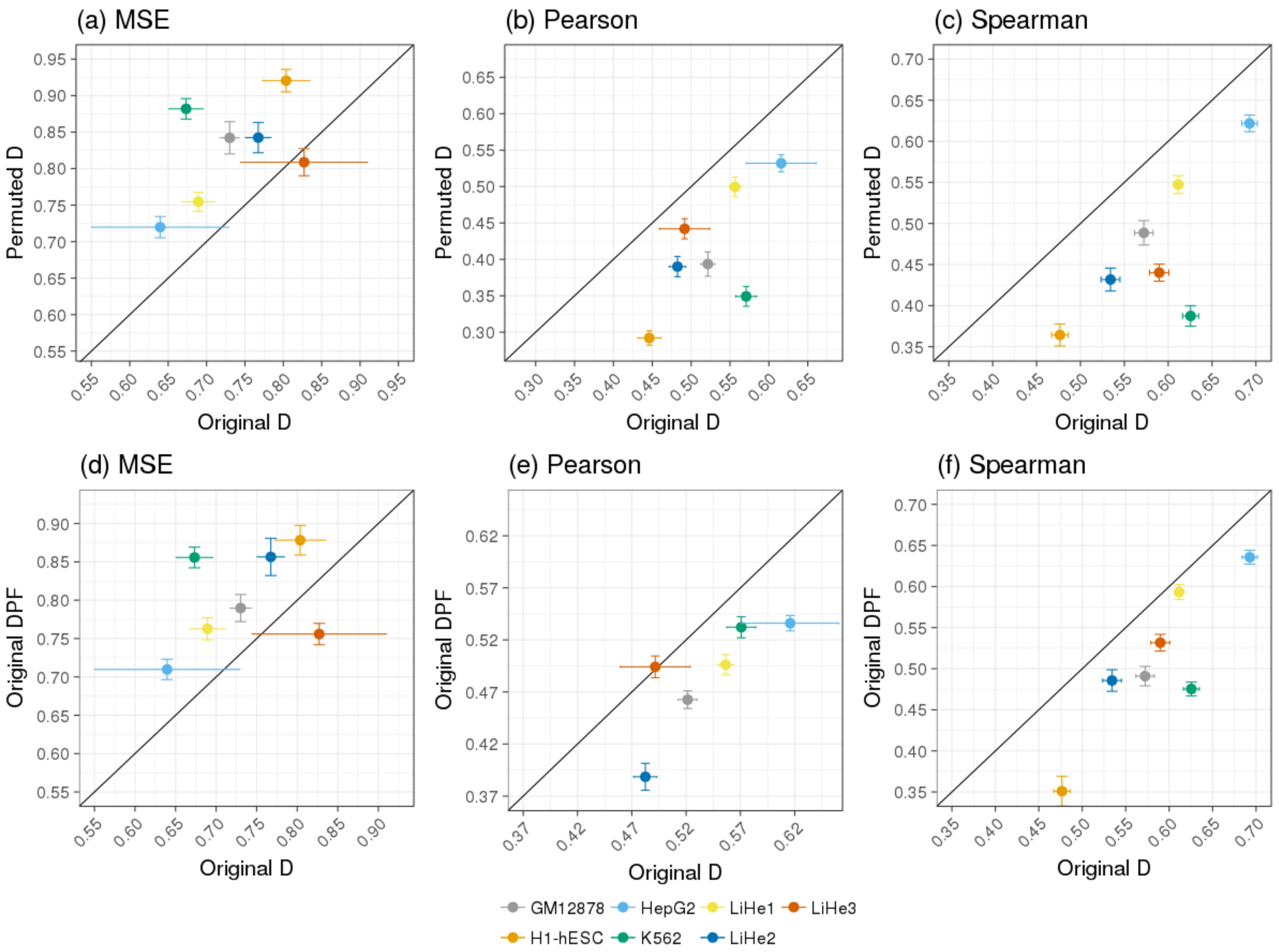

Supplementary Figure 6: Model performance is contrasted for original D and permuted D scores in terms of MSE (a), Pearson correlation (b) and Spearman correlation (c). A comparison of the original D models against models using only peak features (DPF) is shown in (d) using MSE, in (e) using Pearson correlation and in (f) using Spearman correlation. All models use elastic net regression.

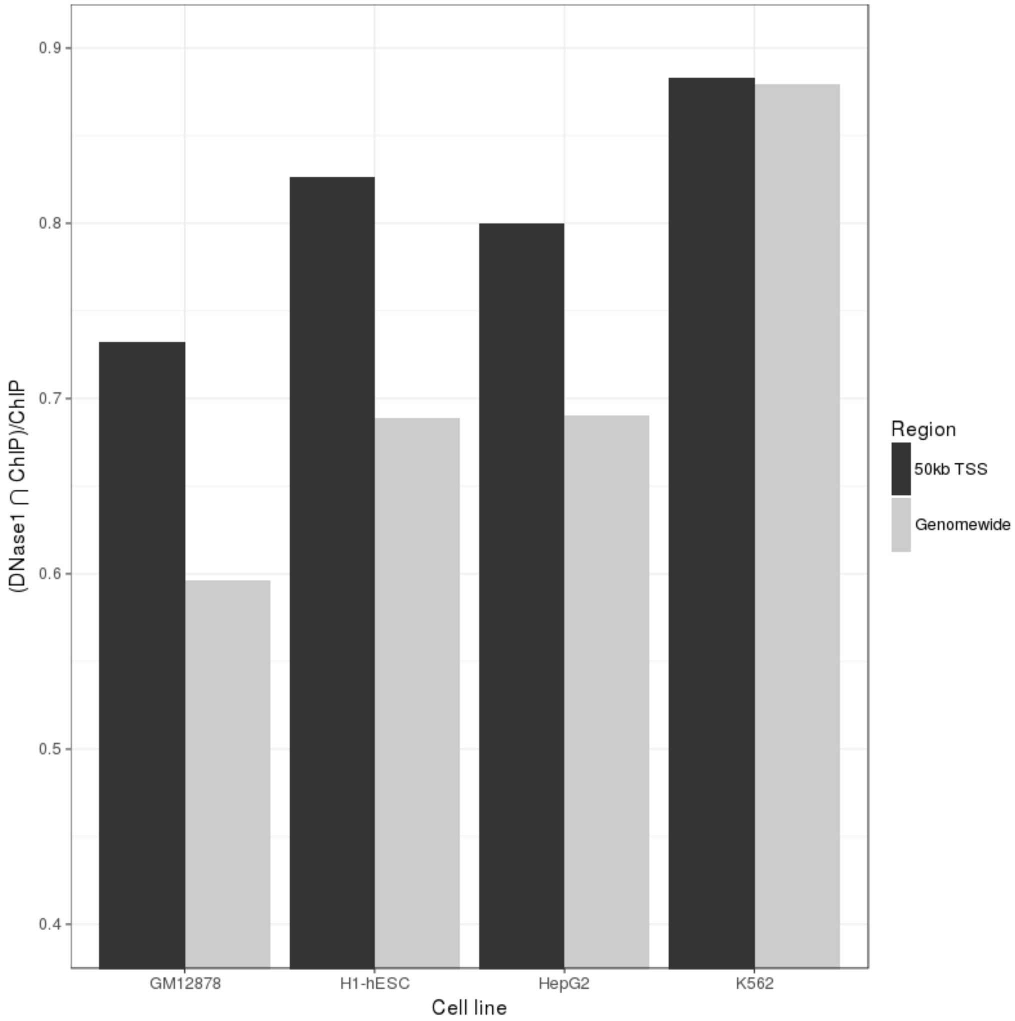

Supplementary Figure 7: Here, the fraction of ChIP-seq peaks that overlap a DNase peak is shown for all ChIP-seq peaks (grey) and for all ChIP-seq peaks in a 50kb window around the 5' TSS of protein coding genes.

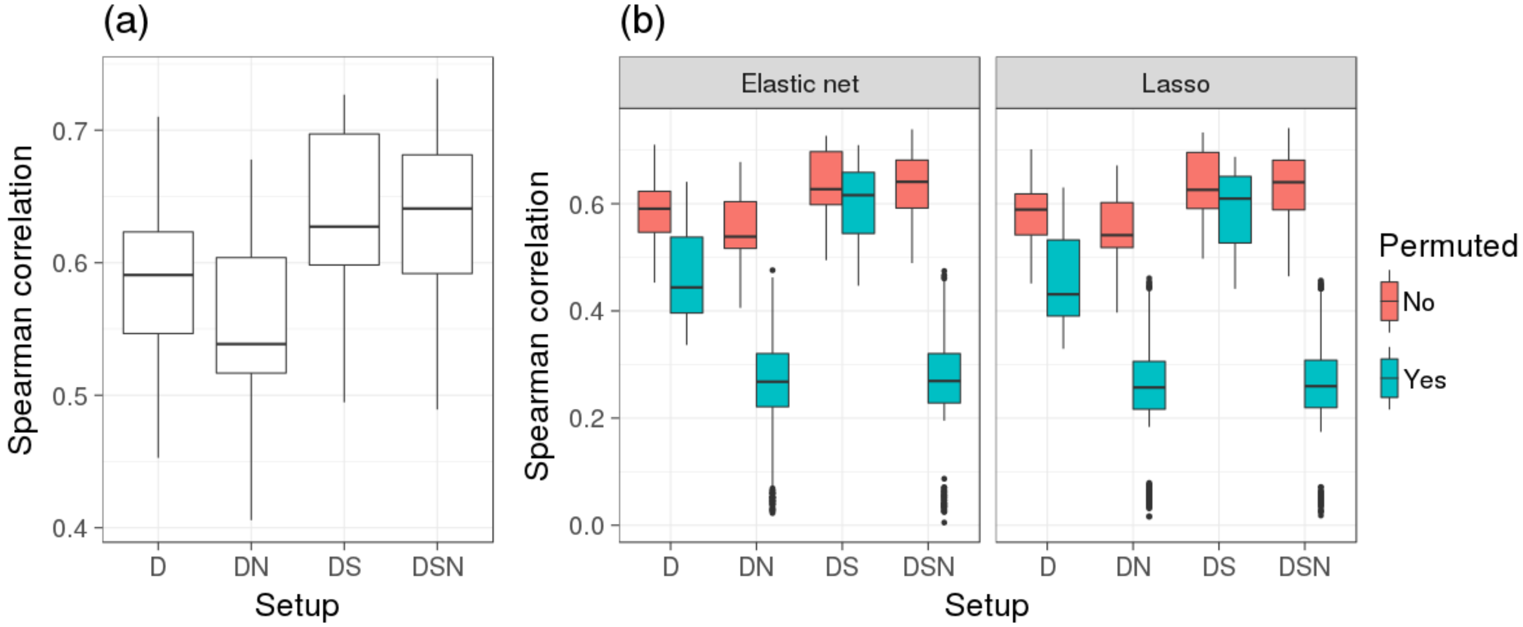

Supplementary Figure 8: In Figure 8a, boxplots show the performance of models using the D, DN, DS, and DSN setup on not permuted data using elastic net. Figure 8b illustrates the effect of different regularization methods. Model performance on permuted and not permuted data is shown for all the D, DN, DS, and DSN scoring schemes using elastic net or lasso regularization.

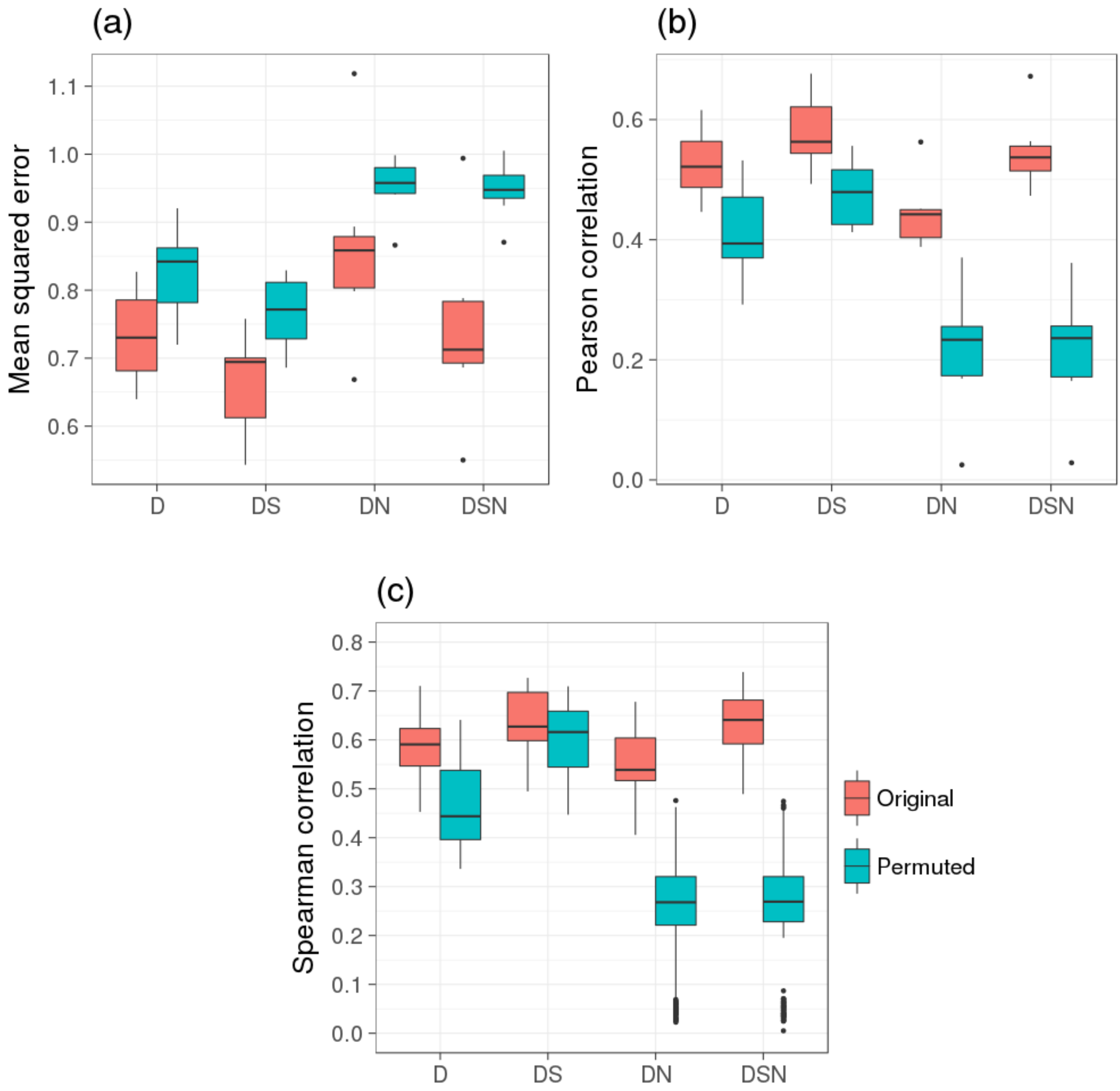

Supplementary Figure 9: Here, the mean squared error for elastic net models based on predicted TFBS sites is shown for original and permuted data using the D, DS, DN and DSN setups, compared using MSE in (a), Pearson correlation in (b) and Spearman correlation in (c).

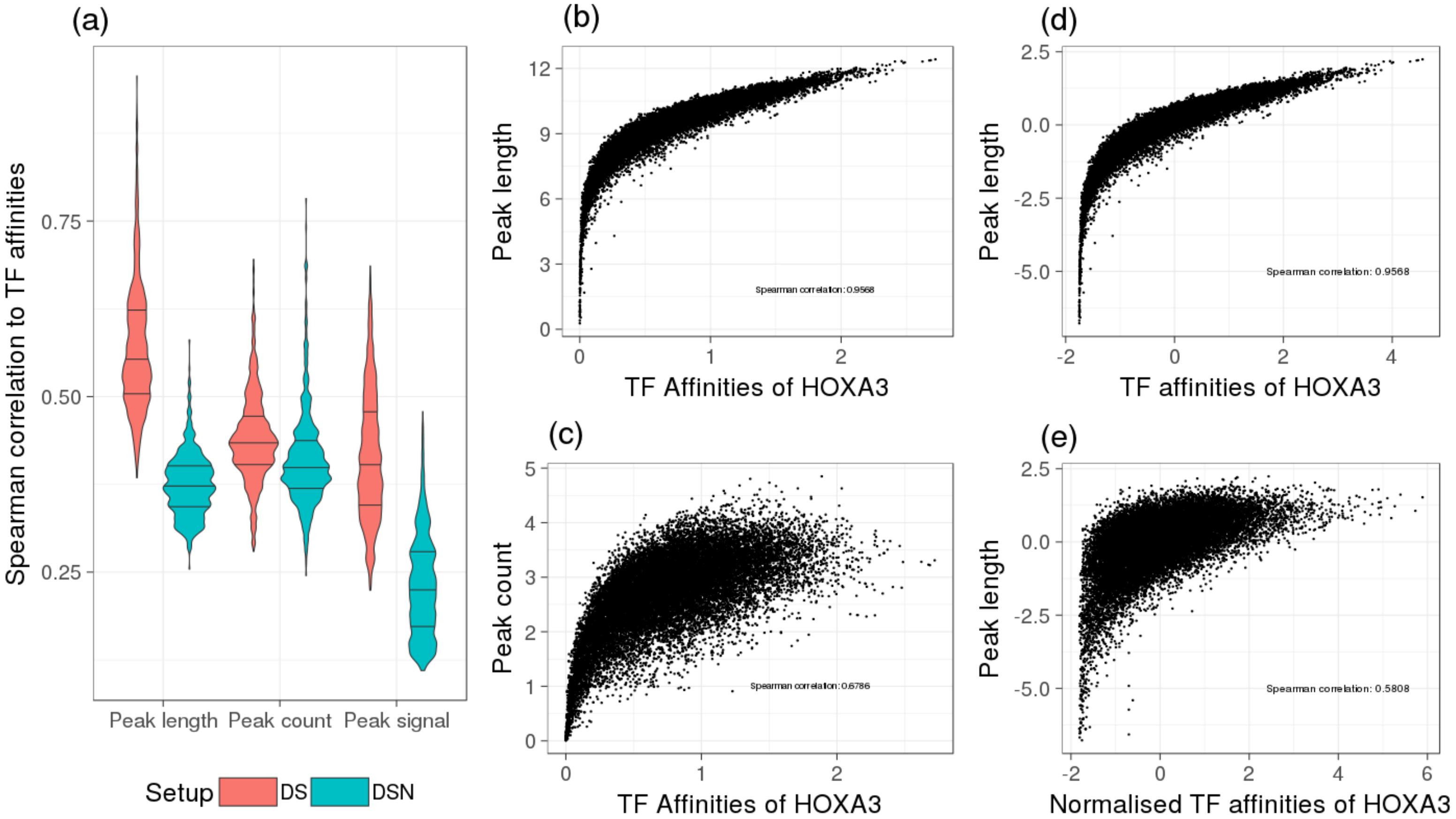

Supplementary Figure 10: (a), we show the pairwise spearman correlation of TF affinities using the DS and DSN scores against *Peak length, Peak count* and *Peak signal*. (b) shows the correlation of TF affinities for HOXA3 using the D setup against *Peak length*. (c) shows the correlation of TF affinities for HOXA3 using the D setup against *Peak count*. (d) shows the correlation of TF affinities for HOXA3 using the DS setup against *Peak length*. (e), the correlation of HOAX3 against *Peak length* is shown using the DSN setup. As Peak length is identical for D and DS, (b) and (d) look alike.

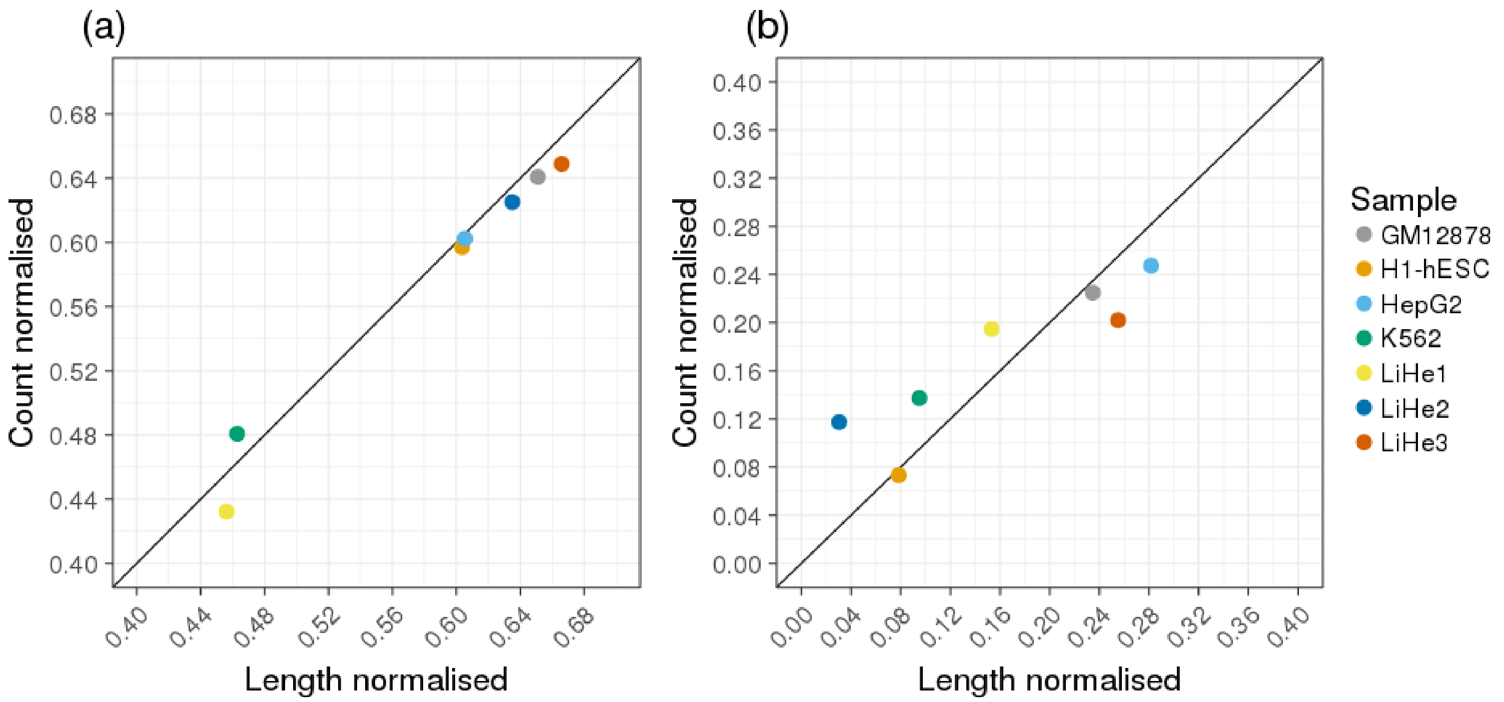

Supplementary Figure 11: (a) Shows the performance of length normalized scores (DN) compared to an additional count normalization (dividing DN scores by the number of considered peaks). (b) Shows the same comparison on permuted data. Model performance is assessed in terms of Spearman correlation. Here, elastic net regularization is used. Error bars are omitted due to a neglectable error.

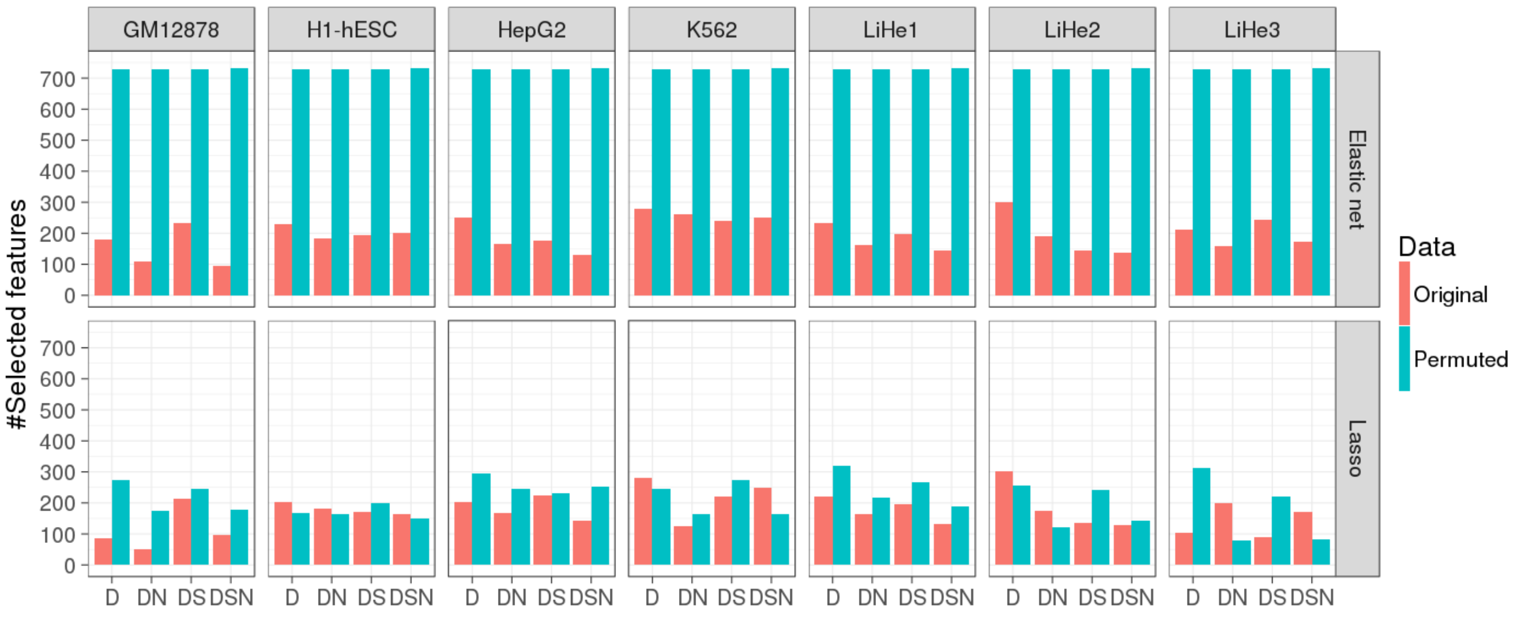

Supplementary Figure 12: The bar plots show the number of selected features (i.e. features with a regression coefficients unequal to zero) per sample using the D, DN, DS, DSN setup with either elastic net or lasso regularization.

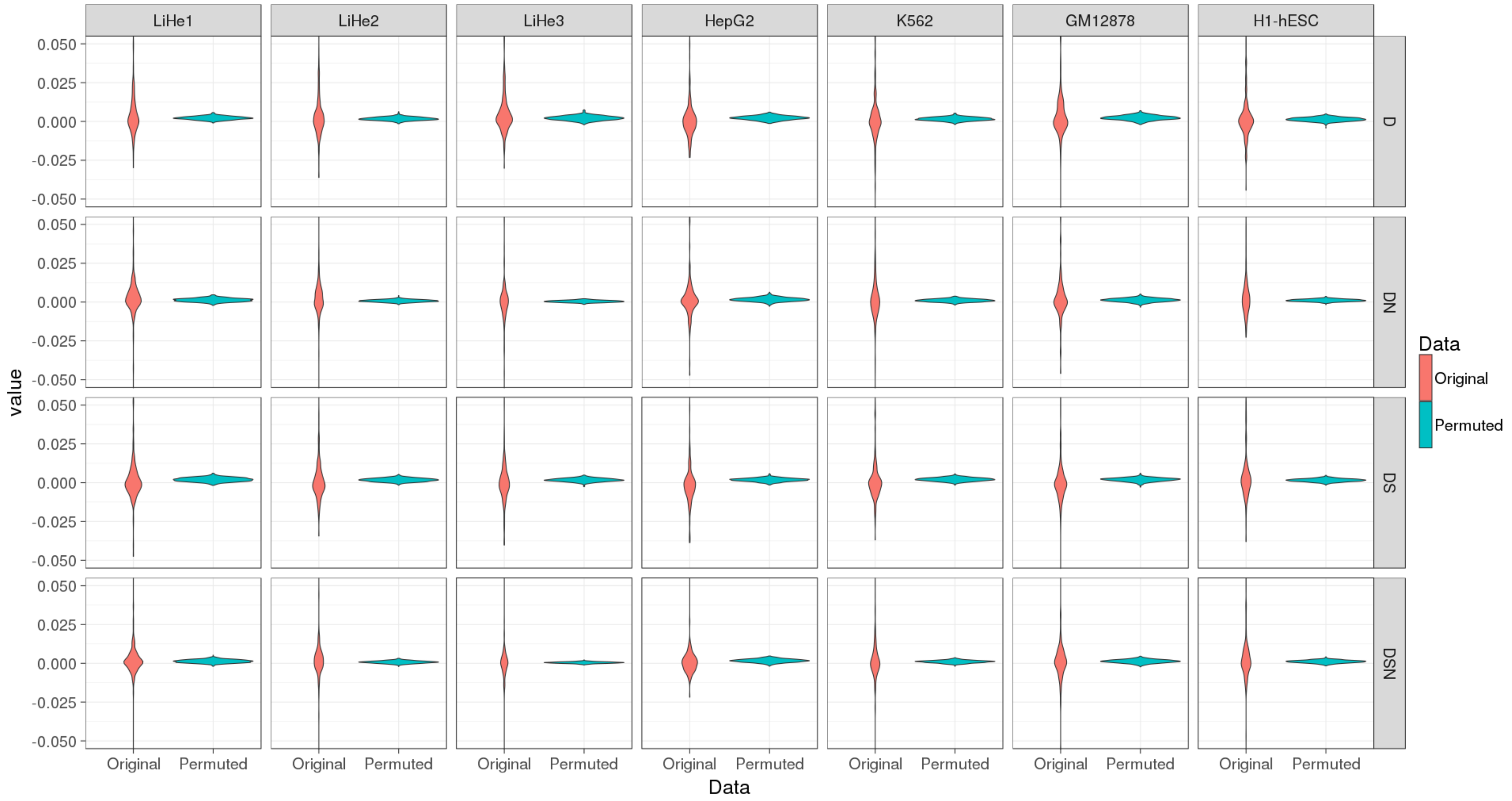

Supplementary Figure 13: Violin plots show the range of regression coefficients per sample inferred on permuted and not permuted data for TF gene scores computed using the D, DN, DS, and DSN setup and elastic net regularization.

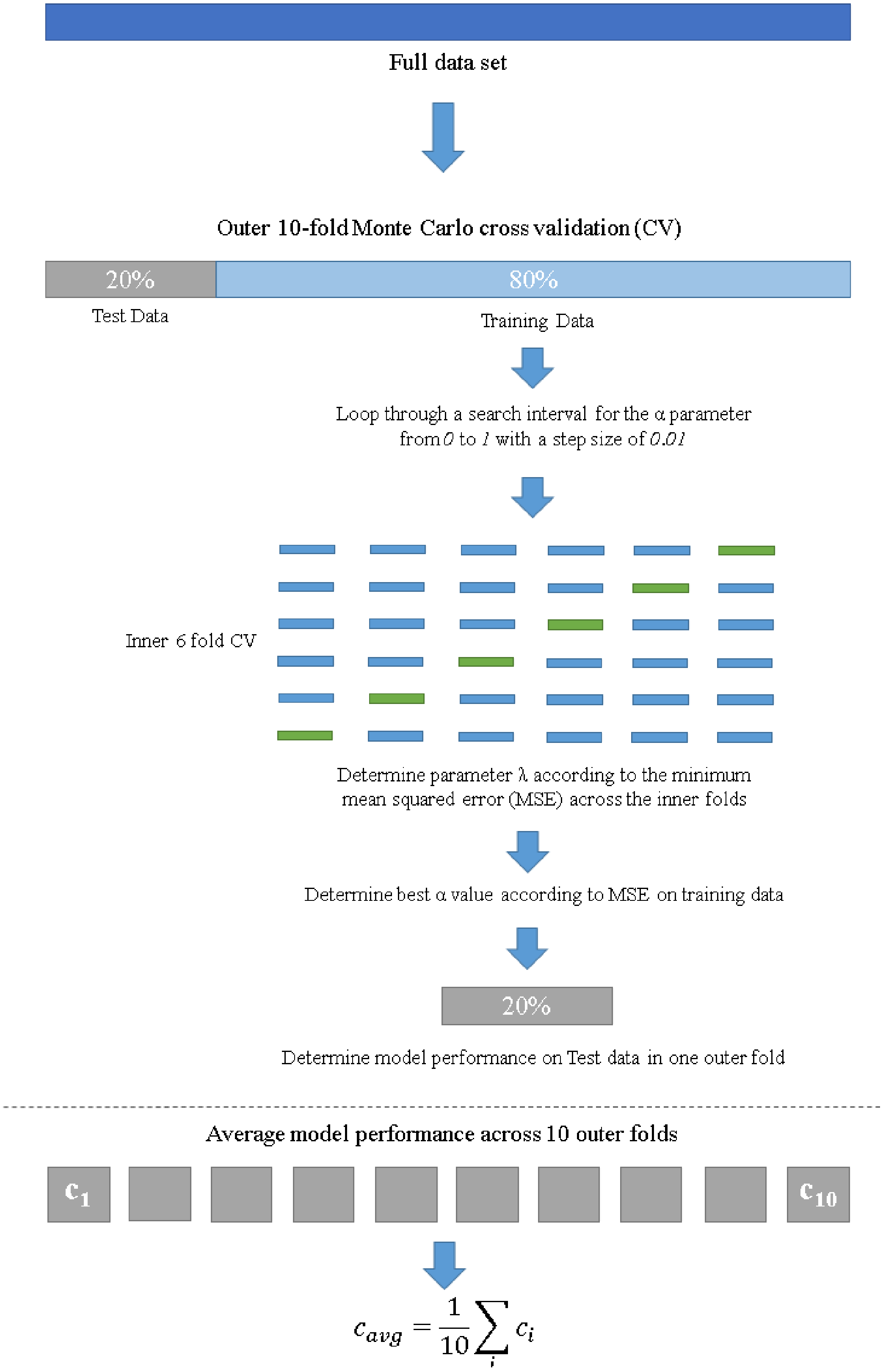

Supplementary Figure 14: Overview of the learning paradigm. We randomly split the original data into Test (20%) and Training (80%) data in a 10-fold outer cross-validation. On the training data, model parameters are learned using a 6-fold inner cross-validation. Model performance is reported as the average performance on the test data across the 10 outer folds.

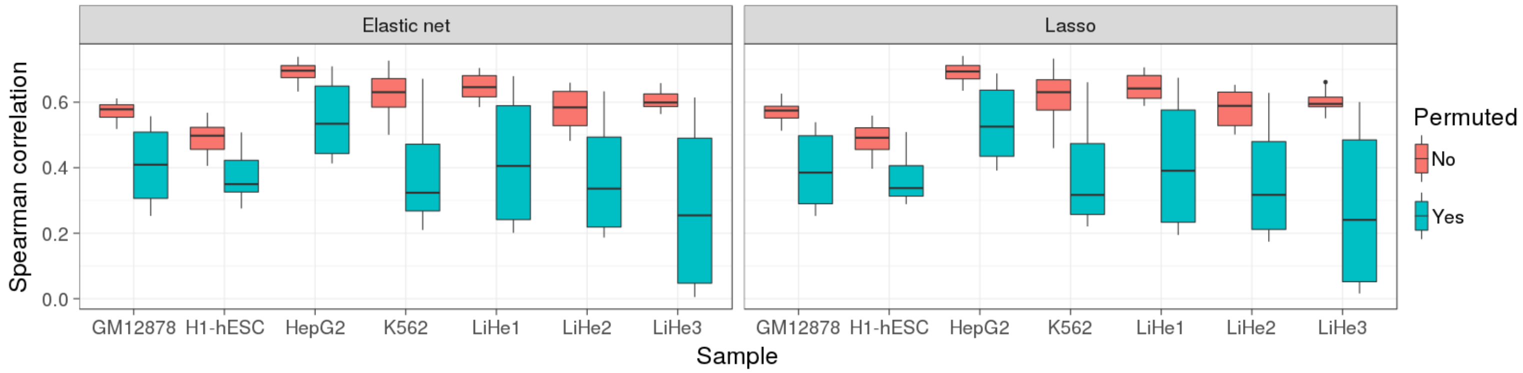

Supplementary Figure 15: Model performance using the DN setup on permuted and not permuted data using either elastic net or lasso regularization is shown per sample.

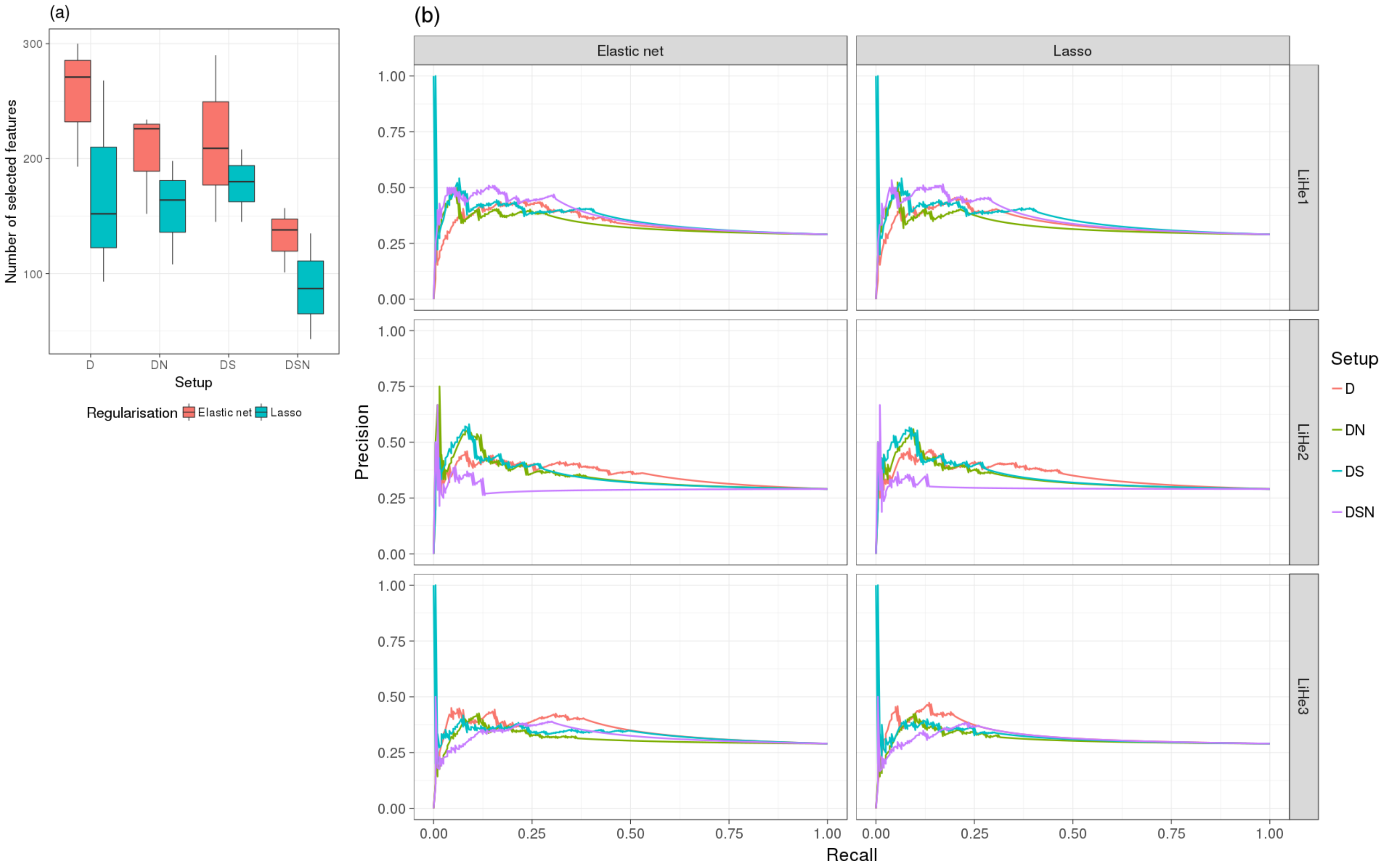

Supplementary Figure 16: (a) shows the number of selected features across the three samples (LiHe1, LiHe2, LiHe3) for the D, DS, DN, and DSN setup using both elastic net and lasso regularization. (b) Shows Precision Recall curves computed using the *PRROC* package distinguishing between samples, annotation setups, and regularization methods.

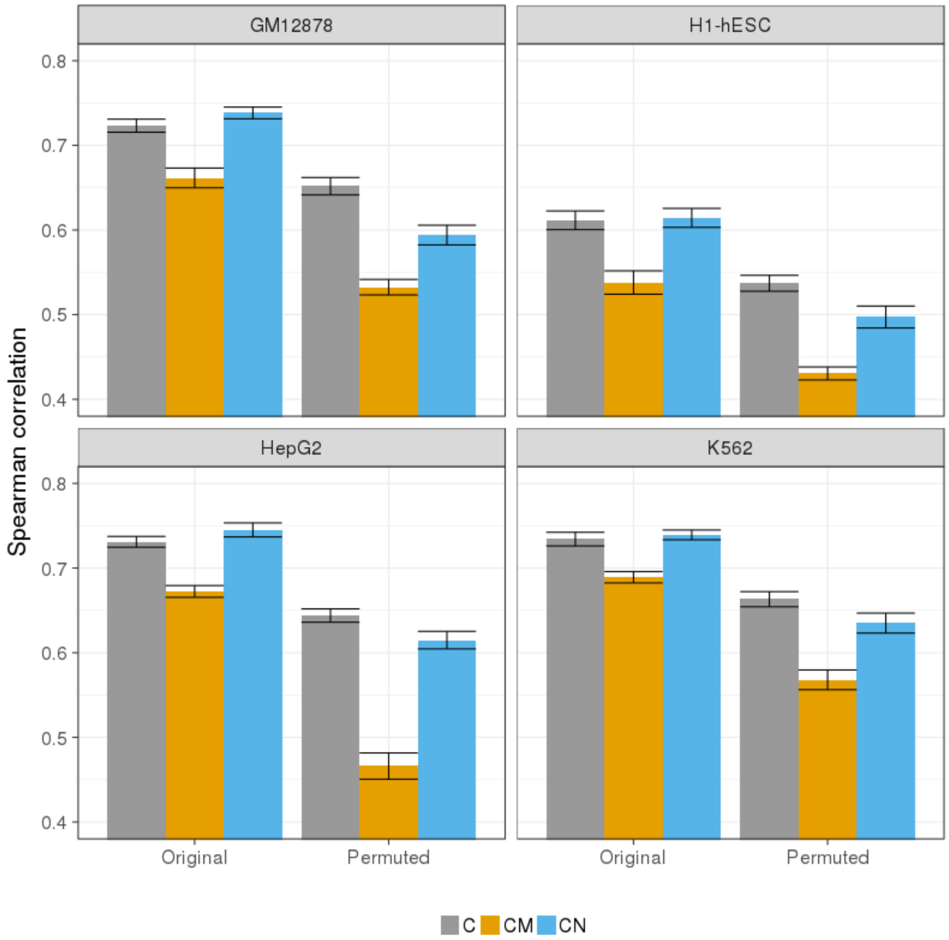

Supplementary Figure 17: Comparison of expression models using elastic net regularization between C, CN, and CM scores using permuted and not permuted data. CM scores refer to a feature matrix which is normalized according to the maximum entry per row. On original data, CM scoring performs worse than both C and CN scores. At the same time, it achieves a worse prediction performance on permuted data than both C and CN scores.

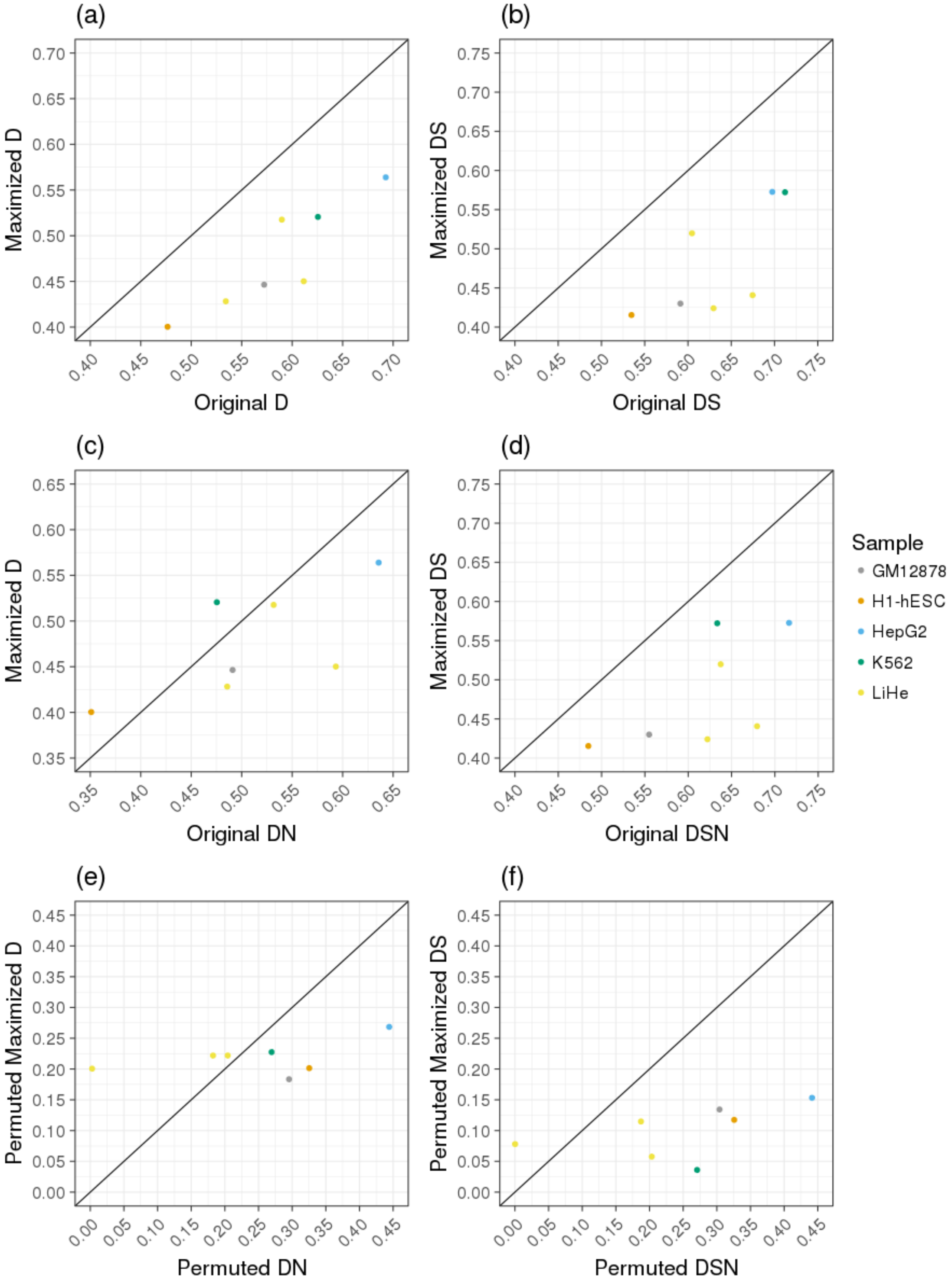

Supplementary Figure 18: Comparison of expression models using elastic net regularization and Spearman correlation for various predicted TF-gene scores. (a) Compares original D scores against Maximized D scores, (b) compares original DS scores against maximized DS scores, (c) contrasts original DN scores against maximized D scores, and (d) reflects the difference between original DSN and maximized DS scoring. In (e) and (f), we compare DN versus maximized D and DSN versus maximized DS on permute data respectively. Error bars are omitted due to a neglectable error.
